# Experimental swine models for perforator flap dissection in reconstructive microsurgery

**DOI:** 10.1371/journal.pone.0266873

**Published:** 2022-04-11

**Authors:** Alexandru Nistor, Lucian P. Jiga, Gratian D. Miclaus, Bogdan Hoinoiu, Petru Matusz, Mihai E. Ionac

**Affiliations:** 1 Division of Microsurgery, Pius Branzeu Center for Laparoscopic Surgery and Microsurgery, Victor Babes University of Medicine and Pharmacy, Timisoara, Romania; 2 Department of Plastic, Aesthetic, Reconstructive and Hand Surgery, Evangelisches Krankenhaus Oldenburg, University of Oldenburg, Oldenburg, Germany; 3 Neuromed, Timisoara, Romania; 4 Division of Clinical Practical Skills, Victor Babes University of Medicine and Pharmacy, Timisoara, Romania; 5 Department of Anatomy, Victor Babes University of Medicine and Pharmacy, Timisoara, Romania; Medical University of Graz, AUSTRIA

## Abstract

**Background:**

Perforator flaps account for a fraction of reconstructive procedures despite their growing popularity. Specific microsurgical skills are required for successful harvesting of perforator flaps, which are difficult to attain through direct operating room training. Cadaver and small animal dissection cannot simulate human perforator dissection, lacking either bleeding and vessel feedback or providing too small calibers. Thus, we have developed and refined over the last ten years five perforator flaps models in living pig, described their harvesting technique and provided evidence for their effectiveness as perforator flap training models.

**Method:**

CT angiography data from ten living pigs was used for detailed examination of the integument’s vascular anatomy. Microsurgical techniques were used to standardize and harvest the perforator flaps in acute models. The same operator-assistant team, with no prior perforator flap harvesting experience, raised all flaps in a sequential manner, one animal per day, during a 7 weeks timespan. Porcine flaps were compared to human counterparts in terms of vessel caliber, dissection times. Immediate flap survival was measured as duration of perforator pulsation after completion of flap harvesting, measured every 10 minutes for up to two hours.

**Results:**

Five perforator flaps were standardized, based on the deep cranial epigastric, thoracodorsal, lateral intercostal, cranial gluteal and dorsal cervical arteries and the operative technique was described in detail. Mean pig perforator size was 1.24±0.36 mm and mean pedicle diameter was 2.78±0.8 mm, which matched closely the human calibers for each flap. Total harvesting time increased 22.4% between the first two experiments due to a more cautious approach following the lack of perforator pulsation in all flaps in the first experimental animal. A total decrease of 44.4% harvesting time between second and last experiment was observed, as expected with all repetitive surgical procedures. Post-operative perforator pulsation time revealed a steep learning curve, with no or short-term pulsatile perforators in the first five pigs, followed by a 275% increase in total perforator pulsation time between 5^th^ and 6^th^ experimental animal. Based on these findings we provide a description of the most common mistakes, their consequences and gestures which can be trained using the pig perforator flaps, in order to overcome these mistakes.

**Conclusion:**

These five pig perforator flap models provide a fast and efficient learning tool to develop perforator flap harvesting skills safely. Surgical training using these five experimental models offers a similar hands-on perforator flap dissection experience as with human tissue, based on the similar sized calibers of both perforators and pedicles with their human counterparts.

## Introduction

Simulating complex surgical techniques in living animal models provides a safe alternative to operating room training, while maintaining a realistic environment in which the key steps of a surgical procedure can be accurately reproduced [1].

Perforator flaps have become commonplace in reconstructive microsurgery and allow us to cover wounds in the head and neck, breast, trunk, upper and lower limbs, that would otherwise have no alternative local choices [2]. Nevertheless, perforator flaps are time-consuming and delicate procedures, requiring thorough post-operative monitoring. Therefore, shortening the duration of perforator flap surgery and lowering the likelihood of flap failure is critical for both patient safety and prevention of secondary morbidity [3].

Repeatedly performing complex perforator flap surgical procedures has been shown to decrease the flap harvesting duration. Total flap harvest time for the deep inferior epigastric (DIEP) flap measures, on average, 54.8 minutes for experienced surgeons, 98.3 minutes for a senior fellow working with faculty (79.3% increase) and 178.8 minutes for a supervised chief resident (226.27% increase) [4]. For a resident with no experience in dissecting perforator flaps, this number is impossible to determine due to ethical constraints, as it would pose a major safety risk to the patient’s surgical outcome [5].

As with all free flap reconstructions, a low failure rate for perforator flaps can be achieved by means of either extensive training under supervision in the operating room (OR), or by simulating the key surgical steps using *in-vivo* animal models [6]. Both options involve a trial-and-error component, which is acceptable only in the animal model scenario.

Although microvascular free tissue transplants are performed now on a daily basis in most major medical institutes around the world, specific training is still mandatory for residents and specialists alike, due to the ethical limitations of operating room training.

This, coupled with the constant decrease in OR exposure due to a worldwide shortening of the residents’ working hours,[7] makes the *in-vivo* training option a go-to destination for supplemental training in reconstructive microsurgery [1].

Traditionally, perforator flap dissection skills were mastered on fresh human cadavers [8]. where surgeons learn the relevant anatomy with direct clinical application. However, the use of cadaver models implies major drawbacks such as limited availability, lack of direct feedback from bleeding tissues or expensive preparation and disposal [8].

As a result, experimental models in small living animals were proposed to simulate the human perforator flaps;[9] several models described in rats [10, 11] suffer from small perforator calibers, requiring supramicrosurgical techniques and instruments. Unlike rodents, pigs have matching vessel sizes,[12] making pigs the ideal choice for surgical training models [13].

We hypothesized that by using *in-vivo* large animal models which accurately simulate the key surgical techniques in human perforator flaps, we can reduce the steepness of perforator flap harvesting learning curve.

Using a multidetector-row computer tomography (MDCT) angiography[14] of the pig’s perforasomes and microsurgical dissection, we describe 5 perforator flaps with constant vascular anatomy and vessel calibers comparable to human flaps, describe the harvesting technique and measure the flap harvesting times and perforator viability. By plotting the learning curves, we provide evidence that these models can be used to overcome the steepness of the perforator flap learning curve.

## Methods

### Experimental study design

Ten pigs (*Sus scrofa domesticus*) of Large White breed (male:female = 3:7), weighing 33±2.31 kg (Smithfield Prod., Timisoara, Romania) were mapped via MDCT and operated sequentially, one animal per day, during a 7 weeks timespan. No control group was required for the standardization of experimental flap models. Experimental procedures were conducted according to the Victor Babes University of Medicine and Pharmacy’s Ethical and Animal Care Committee approval no. 13008. All animals were treated according to the *Policy for the Use of Animals in Teaching and Training* recommended by the Federation of European Laboratory Animal Science Associations (FELASA) [15]. All data points collected from the ten experimental animals were included in the performed statistical analysis.

### Anesthesia

Intramuscular sedation with 2mg/kg xylazine (Rompun®, Bayer HealthCare, Germany) + 10mg/kg ketamine hydrochloride (Calypsol®, Richter Gedeon, Hungary) was followed by endotracheal intubation and ventilation via veterinary anesthesia machine.

Total intravenous anesthesia was induced with 6.6 mg/kg propofol (Lipuro®, B.Braun Melsungen, Germany) and maintained with 90 mg/h propofol. One jugular vein was catheterized using an 18-gauge angiocath needle (Certofix® Trio, B.Braun Melsungen AG, Germany). Blood pressure was recorded via invasive arterial catheter and blood pressure monitor (Bionet BM5, Bionet Co. Ltd, Seoul, Korea). Core temperature was monitored with an intrarectal probe and skin temperature using non-contact infrared thermometer (Fluke 62 Max, Fluke Corporation, Everett, USA). Euthanasia was conducted under full anesthesia at the end of surgical procedures with 0.3ml/kg T61 (MSD Animal Health, Intervet International B.V. Netherlands) and experimental animals were disposed as medical waste.

### AngioCT study

Perforator vessels were measured in all experimental animals via MDCT angiography using a 64-detector scanner (Somatom® 64, Siemens, Germany) with 1 mm slice thickness. Perforator and pedicle caliber measurements were performed on maximum-intensity-projection images in each animal for all perforator flaps. All vessel dimensions are expressed as mean + standard deviation (STDEV). Three-dimensional volume-rendering (3D-VRT) provided a 3D-model for flap localization (see S1 Video, 3D-VRT pig vascular network model and perforator flaps localization).

### Perforator flap planning and dissection

All experimental surgical procedures were carried out at the Pius Branzeu Center for Flap Surgery and Microsurgery. Within the animal house, the pigs were allowed one day of acclimatization before surgery. After induction of anesthesia, the experimental animal was placed on a veterinary surgical table in either dorsal, lateral or ventral decubitus, with limbs extended and secured to the operating table railing.

Doppler sonography confirmed the location of perforators identified prior in the pigs via MDCT.

The same operator-assistant team, with no prior perforator flap harvesting experience, raised all flaps in a sequential manner, one animal per day, during a 7 weeks timespan.

Flap dissection was performed under 4x loupes magnification ensuring adequate visualization of the perforator vessels and allowing for careful handling of the soft tissues. Emphasis was placed on using atraumatic dissection techniques, with skin incision performed only with the cold knife and hemostasis performed only with soft bipolar coagulation. The same training setting was used throughout all experimental animals.

Time to completion of flap harvesting was recorded for each flap in each animal.

Due to ethical constrains, no long-term follow-up of the flaps was possible. Thus, the duration of perforator pulsation after flap harvesting was chosen to objectively assess the quality of the surgical dissection and flap viability. Perforator pulsation was observed after flap harvesting every 10 minutes, until no longer detectable and duration of pulsation was documented.

### Statistical analysis

The statistical analysis has been performed using SPSS software (IBM SPSS, Armonk, NY:IBM Corp). Data distribution has been considered normal following normality analysis for each outcome, including skewness, kurtosis, and Q-Q plot assessments, as well as Shapiro-Wilk and Kolmogorov-Smirnov tests, and continuous values have been displayed as means ± standard deviation. Paired-samples t-tests were used for perforator and pedicle diameter and duration of perforator flap harvesting time comparison between the five types of flaps. One-way ANOVA was also performed in order to analyze differences between duration of perforator flap harvesting time and learning curve for the intervention. P values <0.05 were considered statistically significant

## Results

### New experimental perforator flaps in pigs

MDCT angiography identified multiple perforasomes with constant anatomy, out of which we selected five perforasomes around four anatomically distinct body areas: ventral abdominal, lateral thoracic, gluteal and dorsal cervical. In these areas, we designed five perforator flaps, four of which simulate already established perforator flaps in humans and one flap without human counterpart. The resulting standardized perforator flaps, described below, were refined during 18 post-graduate University training courses, serving as training models for 180 surgeons from all over the world. *Italic names* represent the current nomenclature according to Nomina Anatomica Veterinaria [16].

1. Deep cranial epigastric artery perforator flap (DCEAPf)

Mimicking the DIEP flap, this model uses perforators arising from the deep cranial epigastric artery (DCEA) (*A*. *epigastrica cranialis profunda*), instead of the deep caudal one, which has 50% smaller perforators. DCEA is centered on the abdominal wall mid-segment, providing a more comfortable surgical workspace, above the umbilicus and urogenital opening in male pigs (See S2 Video, 3D-VRT of DCEAPf flap planning and landmarks).

*Blood supply*. 6–8 perforators form the lateral and medial rows, parallel to the *linea alba* on each hemiabdomen, arising from DCEA, an internal thoracic artery *(A*. *thoracica interna)* terminal branch running underneath the nipple line. Both medial and lateral row perforators are pure muscle perforators, with an oblique course through the *rectus abdominis*. The medial row’s skin projection lies medial to the nipple line and has larger caliber perforators compared to the lateral row.

*Veins*. The superficial cranial epigastric vein (SCEV) *(V*. *epigastrica cranialis superficialis)* can be palpated percutaneously, coursing parallel to DCEA and the two perforators rows, running in between the perforator lines. Two *venae comitantes* of similar diameter to the arterial perforator assure adequate venous drainage if undamaged.

*Pedicle length*. With a longer pedicle (11.36 ± 1.9 cm) and due to the ease of harvest of the DCEA and vein, this flap allows for coverage reaching well beyond the recipient artery.

*Flap design*. In dorsal decubitus, with caudal limbs secured to the operating table, an elliptical flap is planned, centered between the second and fourth nipple line, which can be sub-divided in two 40cm^2^ half-ellipses with a 10 cm-long base centered on the *linea alba* and around two nipples. This design assures inclusion of large perforators, eliminating pre-op Doppler sonography (see S2 Video, 3D-VRT of DCEAPf flap planning and landmarks).

**Fig 1:**
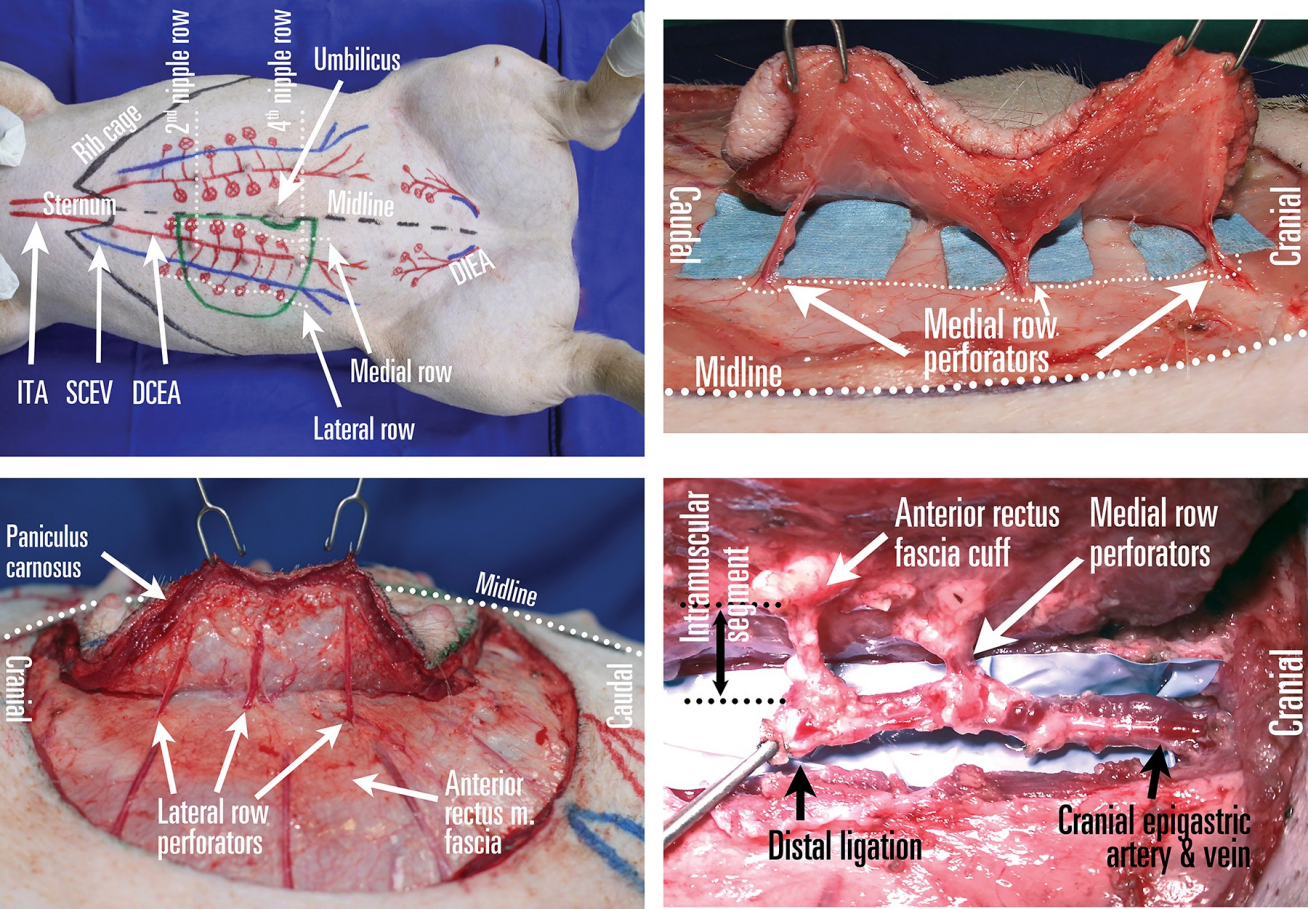
Deep cranial epigastric artery perforator flap planning in pigs. (A) DCEAPf planning between the 2^nd^ and 4^th^ nipple line, centered on the deep cranial epigastric artery (DCEA) and including the medial and lateral row of skin perforators. ITA–internal thoracic artery; SCEV–superficial cranial epigastric vein; DIEA–deep inferior epigastric artery. (B) Dissection of lateral row of perforators. (C) Dissection of medial row perforators. Dotted line marks the incision along the anterior rectus muscle sheet. (D) Epigastric artery dissected and transected distal to the skeletonized perforators, with no sign of vessel spasm. Republished from personal archive under a CC BY license, with permission from Dr. Nistor Alexandru, original copyright 2016.

**Operative technique** (Fig 1): Skin incision advances from the half-ellipse’s lateral margin towards medial, up to the cranial SCEV, which is dissected out and used to supercharge the flap. Transecting the *panniculus carnosus* maintains the correct dissection plane above *rectus abdominis*’s anterior fascia. Lateral row dissection progresses from lateral towards *linea alba*, preserving these perforators until a larger medial one is found. Exposing the medial perforator row is performed from the *linea alba* towards lateral. After 2–3 main medial perforators are found, the *rectus abdominis* anterior sheet is incised, followed by subfascial and, most critically, intramuscular dissection of the perforators, with an oblique intramuscular course. A blood free operating field is essential. Perforator pulsation is used to follow the vessel towards DCEA pedicle, where artery and vein are transected distal to the chosen perforator. The DCE pedicle is dissected at the desired length out of the *rectus abdominis*, ligating any additional proximal perforating branches.

**Mean harvesting time** was 101.6±27.97 min, with the first two flaps requiring 93 and 158 min and the last two 84 and 76 min.

**Perforator flap pulsation** was observed for more than 120 min in the last two experimental animals, compared to no pulsation after flap harvesting in the first two pigs.

1. Thoracodorsal artery perforator flap (TDAPf)

TDAPf has a thinner, more pliable skin paddle compared to DCEAPf.

*Arteries*. The main perforator arises perpendicular between *latissimus dorsi*’s cranial and ventral border muscle fibers, originating from the transverse thoracodorsal artery *(A*. *thoracodorsalis pars transversalis)*. The thoracodorsal artery enters the *latissimus dorsi* on its posterior surface, dividing at 45° into descending and transverse branches, the latter continuing its course caudally towards dorsal margin of *latissimus dorsi*.

*Veins*. A similar-sized vein along the perforating artery feeds into the transverse thoracodorsal vein.

*Pedicle length*. The pedicle can be harvested up to the subscapular artery, with a mean length of 8.22±2.15 cm.

*Flap design*. Within lateral decubitus, abduct the thoracic limb for easier axillary dissection. Using Doppler sonography, the main perforator is found along *latissimus dorsi*’s cranial border, before submerging under the trapezius muscle *(M*. *trapezius pars thoracica)*, 5–6 cm caudal from the scapula’s spinous tuber *(Tuber spinae scapulae)*, running towards the midaxillary line *(Linea axillaris media)*. An oval flap 10 cm across (50 cm^2^) is centered on the perforator’s projection, the long axis running dorso-caudal towards the axilla (See S3 Video, 3D-VRT pig hemithorax model, depicting TDAPf positioning).

**Fig 2:**
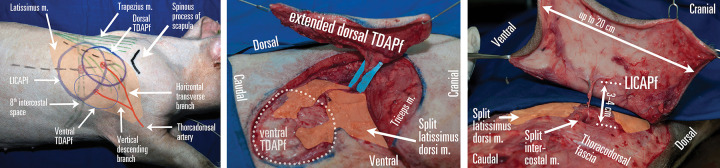
Thoracodorsal artery perforator flap (TDAPf) and lateral intercostal artery perforator flap (LICAPf) planning in pigs. (A) TDAPf can be harvested with a dorsal or ventral skin paddle, based on either the horizontal transverse branch or the vertical descending thoracodorsal artery branch. In both cases, the flap extends in the territory irrigated by the lateral intercostal perforators, which pierce the *latissimus dorsi* on their way to the skin and can be mistaken for the TDA perforator. (B) An extended TDAPf harvested by combining the dorsal and ventral TDAP flap paddles. The *latissimus dorsi* muscle has been split to allow for a longer pedicle, which can be extended up to the thoracodorsal artery for a total length of 12–14 cm. (C) A large LICAPf can be harvested if no TDAPf is planned on the same hemitorax. Both latissimus dorsi and intercostal muscles are split to allow for a longer pedicle. Republished from personal archive under a CC BY license, with permission from Dr. Nistor Alexandru, original copyright 2016.

**Operative technique** (Fig 2A and 2B): The caudal flap tip is dissected above *latissimus dorsi*’s anterior fascia, transecting the *panniculus carnosus*. Main perforator is found half-way towards the cranial flap tip, with secondary perforators found caudally. The anterior fascia is incised, main perforator is dissected in its course parallel to *latissimus dorsi*‘s muscle fibers up to the thoracodorsal artery, which can be further dissected up to the subscapular artery, allowing for the longest intramuscular dissection.

**Mean harvesting time** was 107.5±20.13 min, with the first two flaps requiring 106 and 160 min and the last two 86 and 72 min.

**Perforator flap pulsation** was observed for more than 90 min in the last two experimental animals, compared to 20 min pulsation after flap harvesting in the second experimental animal.

2. Lateral intercostal artery perforator flap (LICAPf)

This flap simulates the human ICAPf, described 1979,[17] with three variations according to skin island location (dorsal, lateral and anterior).

*Arteries*. Three branches divide the intercostal arteries (ICA) *(Aa*. *intercostales dorsales)* in three segments: dorsal *(Ramus dorsalis)*, spinal *(Ramus spinalis)* and medial branch *(Ramus cutaneus medialis)*. The ICA continues with the lateral segment, giving rise to the lateral skin perforators *(Rami cutanei laterales)*, on which the LICAPf is based. Every intercostal space contains one ICA; the perforator arising from the 8^th^ intercostal space has the largest caliber. ICA anastomoses terminally with the ventral intercostal branches *(Rami intercostales ventrales)* from the internal thoracic artery *(A*. *thoracica interna)*.

*Veins*. One perforating vena comitante drains into the dorsal intercostal vein *(V*. *intercostalis dorsalis)*, draining the intervertebral vein as well, supplying the left azygos vein *(V*. *azygos sinistra)* and anastomoses ventrally with the corresponding ventral intercostal vein *(V*. *intercostalis ventralis)*.

*Pedicle length*. This flap has the shortest pedicle of all five experimental models, due to the position of the ICA on the posterior wall of the ribcage. Even so, a pedicle long enough to be anastomosed can be harvested, with an average length of 2.58± 0.59 cm.

*Flap design*. Within lateral decubitus, secure the cranial limbs in slight extension. Mark the scapula’s spinous tuber *(Tuber spinae scapulae)* and draw a longitudinal line running cranio-caudal from this landmark. LICAP is consistently found in the 8^th^ intercostal space. Doppler sonography is optional. Draw a 10x8cm ellipse (88cm^2^) with the long axis running dorso-ventral, centered on the perforator (See S4 Video, 3D-VRT pig abdomen model, depicting LICAPf planning).

**Operative technique** (Fig 2C): Ventral flap margin dissection is performed above the anterior fascia of *latissimus dorsi*, transecting the *panniculus carnosus*. If Doppler sonography has not been used, dissect with caution and find the main perforator in the flap’s mid-segment, follow it through *latissimus dorsi’*s fibers and into intercostal muscle fibers, spreading the intercostal space. Lateral perforator arises from ICA, running on the rib’s posterior side, which can be sectioned to harvest a composite rib flap or a longer pedicle.

**Mean harvesting time** was 109.8±43.35 min, with the first two flaps requiring 144 and 184 min and the last two 60 and 63 min.

**Perforator flap pulsation** was observed for more than 90 min and 120 min in the last two experimental animals, compared to no pulsation after flap harvesting in the first two pigs.

1. Cranial gluteal artery perforator flap (CGAPf)

Corresponding to the human superior gluteal artery perforator flap (SGAPf), this model provides the thinnest fasciocutaneous paddle.

*Arteries*. A single perforator pierces the middle gluteal muscle *(M*. *gluteus medius)* and the superficial gluteal muscle *(M*. *gluteus superficialis)*, which covers partially the middle one. The perforator courses oblique towards caudal-lateral, arising from the cranial gluteal artery *(A*. *glutea cranialis)*, an internal iliac artery *(A*. *iliaca interna)* branch.

*Veins*. One *vena comitante* follows the perforator and drains into the pedicle’s vein (*V*. *glutea cranialis)*.

*Pedicle length*. This flap has a rather short pedicle, of 6.42±1.54 cm. Due to the depth of the working field, dissection of the pedicle cannot be performed on a longer distance.

*Flap design*. The pig is placed in lateral decubitus, with hind limbs secured in extension and internal rotation. On the hip’s lateral surface, a line uniting the ventro-cranial iliac spine (*Tuber coxae*) with the sacral tuber (*Tuber sacrale)*, close to the sacroiliac joint, marks the gluteal muscle’s cranial border. Using Doppler sonography, the perforator is found caudal to this line, 4–5 cm caudally from the coxal tuberosity. Draw a 10 cm long ellipse (50cm^2^) centered on the perforator, oriented cranio-caudally (See S5 Video, 3D-VRT pig buttock model, depicting CGAPf planning).

**Fig 3:**
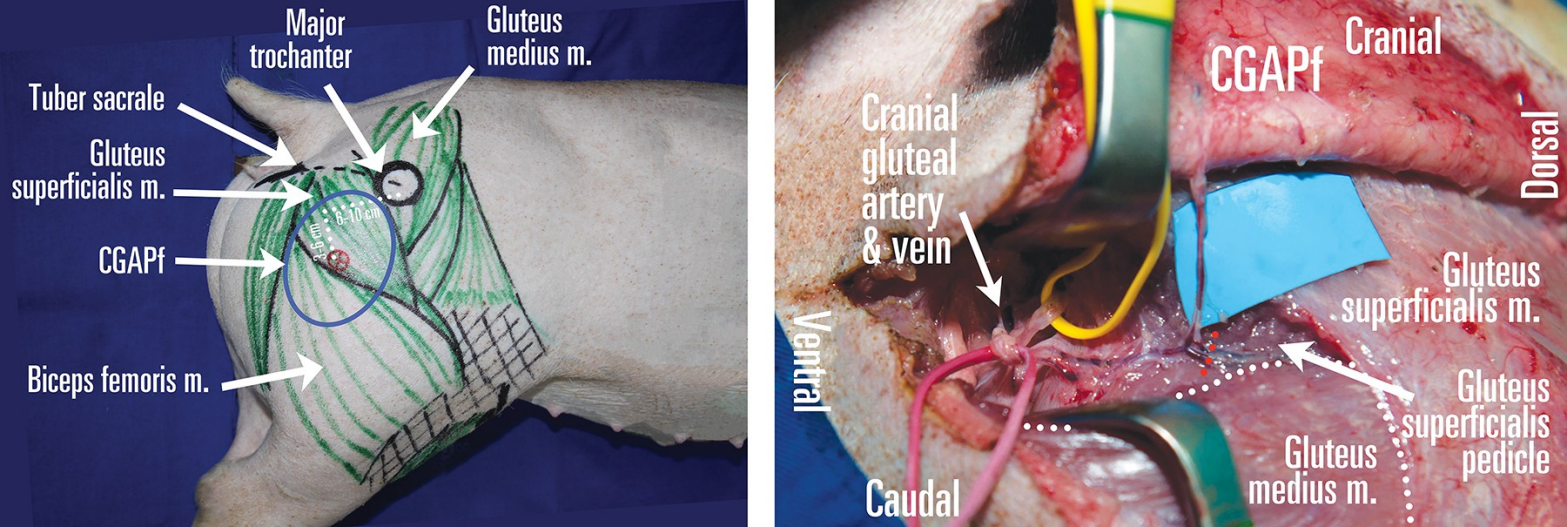
Cranial gluteal artery perforator flap (CGAPf) planning in pigs. (A) Cranial gluteal artery (CGA) perforator pierces first the *gluteus medius* m. followed by *gluteus superficialis* m. on its way to the skin and is consistently located 6–10 cm caudal and 3–6 cm ventral from the major trochanter. (B) The CGA terminal branches are the *gluteus superficialis* m. pedicle and the CGA perforator, which has a long intramuscular course. The *gluteus superficialis* m. pedicle has to be transected (red dotted line) in order to reach the CGA, about 6 cm deep. Republished from personal archive under a CC BY license, with permission from Dr. Nistor Alexandru, original copyright 2016.

**Operative technique** (Fig 3): Incise the caudal flap margin, transecting the very thin *panniculus carnosus*, covering the thin and broad superficial gluteal muscle (SGM), which joins the tensor fascia lata muscle *(M*. *tensor fasciae latae)* on its cranial border and on the caudal edge the gulteo-femoral muscle *(M*. *gluteofemoralis)*.

The perforator is found 6–8 cm caudally from the coxal tuberosity and 4–5 cm ventral from the axial line passing through the coxal tuberosity, with a short segment through the SGM, continuing along the middle gluteal muscle’s ventro-cranial border, with a septocutaneous course between the *Tensor fascia lata* muscle in the superficial layer and the iliac muscle in the deep layer on the caudal side and the middle gluteal muscle on the cranial side, before arising from the CGA. The latter one can be dissected for about 3–4 cm. Further dissection becomes difficult due to the working space depth.

**Mean harvesting time** was 144±23.92 min, with the first two flaps requiring 139 and 177 min and the last two 128 and 112 min.

**Perforator flap pulsation** was observed for more than 90 min in the last two experimental animals, compared to no pulsation after flap harvesting in the first two harvested flaps.

1. Deep cervical artery perforator flap (DCAPf).

With no correspondent in humans, this flap’s accessibility and high density of large perforators render it extremely useful as training model.

*Arteries*. Centered on the 3^rd^-4^th^ cervical vertebrae’s skin projections, the perforators on each side of the middorsal line can be traced back to the corresponding left or right deep cervical artery *(A*. *cervicalis profunda)*, arising from the costo-cervical trunk *(Truncus costocervicalis)*, a branch of the left subclavian artery (*A*. *subclavia sinistra)*. The DCA’s terminal branch is the left first intercostal dorsal artery (*A*. *intercostalis dorsalis I sinistra)*, which should not be mistaken for a perforator.

*Veins*. Each perforator is accompanied by two *venae comitante*, draining in the deep cervical veins alongside the DCA.

*Pedicle length*. This flap presents the deepest working space of the entire five, making the dissection of the pedicle difficult, but nevertheless a 6.42±1.54 cm pedicle was possible to be obtained.

*Flap design*. In ventral decubitus, with head in lateral rotation, secure the front limbs in extension. Palpate and mark the spinous process of the first thoracic and fourth cervical vertebra *(Processus spinousus vertebrae thoracicae I*,*—cervicale IV)*. Using Doppler sonography, identify perforators cranial to this area, divide the dorsal neck region in two halves with a 8cm-long base, centered on the 4^th^ vertebrae, ensuring inclusion of septocutaneous perforators in each 30 cm^2^ half-flap (See S6 Video, 3D-VRT pig dorsal cervical model, depicting DCAPf planning).

**Fig 4:**
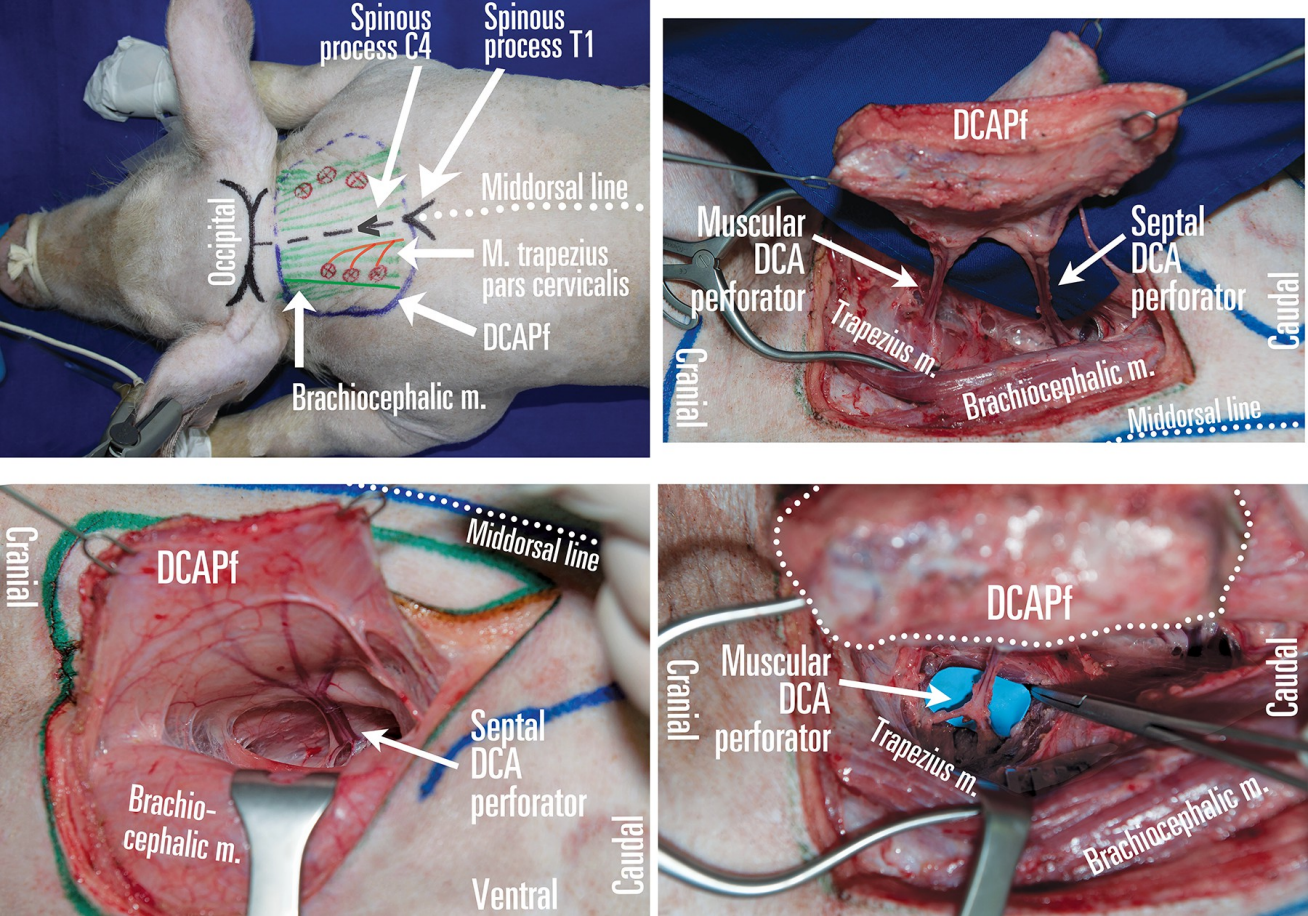
Deep cranial artery perforator flap (DCAPf) planning in pigs. (A) The DCAPf can be designed either as a half-flap, allowing two surgeons to work simultaneously, or as a single extended flap based on one or multiple perforators, which can be either musculocutanous, septocutaneous or both. The spinous process of C4 and T1 vertebrae serve as bony landmarks for flap planning. (B) Dissecting from the lateral margin of the flap over the *brachiocephalic* and *trapezius* m., the septocutaneous perforators are uncovered first, which arise from the DCA. (C) The flap paddle can be vascularized by either muscular or septal DCA perforators, or both. (D) Dissection of the muscular perforator leads to exposure of the deep cranial artery. Republished from personal archive under a CC BY license, with permission from Dr. Nistor Alexandru, original copyright 2016.

**Operative technique** (Fig 4): Dissect the lateral flap margin towards middorsal, transecting the *panniculus carnosus*, above the trapezoid’s anterior fascia (*M*. *trapezius pars cervicalis)* and the brachiocephalic muscle *(M*. *brachiocephalicus)* on the latero-ventral side. 2–3 large perforators can be traced through the trapezoid muscle, before entering the septum between the *splenius capitis* and the *semispinalis capitis* muscles and reaching the DCA, which can be further traced 3–4 cm before the 6–8 cm total operating field depth prevents further dissection towards the costo-cervical trunk. 5–6 cm lateral from the middorsal line, septocutaneous perforators arise between the trapezius and *pars thoracalis* of *latissimus dorsi*.

**Mean harvesting time** was 161.3±19.77 min, with the first two flaps requiring 167 and 149 min and the last two 148 and 119 min.

**Perforator flap pulsation** was observed for more than 120 min in the last two experimental animals, compared to no pulsation after flap harvesting in the first two.

### Vessel calibers

Table 1 provides a descriptive comparison of mean perforator and pedicle diameter as well pedicle length for each flap with the corresponding diameters from human flaps, taken from the literature.

**Table 1:** Comparison between pig perforator flaps and human correspondents.

Pig Perforator flap	Vessel of origin in pigs	Perforator type	Human correspondent	Perforator diameter in pigs	Perforator diameter in humans	Pedicle diameter in pigs	Pedicle diameter in humans	Pedicle length in pigs	Pedicle length in humans
**DCEAPf**	Internal thoracic artery	Muscle	DIEP	1.6 ± 0.3 mm	1.07 ± 0.85 mm[18]	2.3 ± 0.3 mm	3.3 mm[19]	11.33 ± 1.88 cm	16.9 cm[18]
**TDAPf**	Subscapular artery	Muscle	TDAP	0.9 ± 0.2 mm	0.90 ± 0.3 mm[20]	3.8 ± 0.3 mm	2.8 ± 1.2 mm[20]	8.22 ± 2.15 cm	14.0 cm[20]
**LICAPf**	Aorta	Muscle	ICAP	1.7 ± 0.4 mm	0.7 ± 0.2 mm[21]	2 ± 0.4 mm	2.4 ± 0.3 mm[21]	2.58 ± 0.59 cm	3–5 cm[22]
**CGAPf**	Internal iliac artery	Muscle / septal	SGAP	1.5 ± 0.3 mm	0.6 ± 0.1 mm[23]	2.1 ± 0.5 mm	2.17 ± 0.5 mm[24]	6.42 ± 1.54 cm	8.7–9.2 cm[25]
**DCAPf**	Costo-cervical trunk	Muscle / septal	None	1.5 ± 0.3 mm	-	3.7 ± 0.4 mm	-	7.73 ± 2.34 cm	-

**Fig 5:**
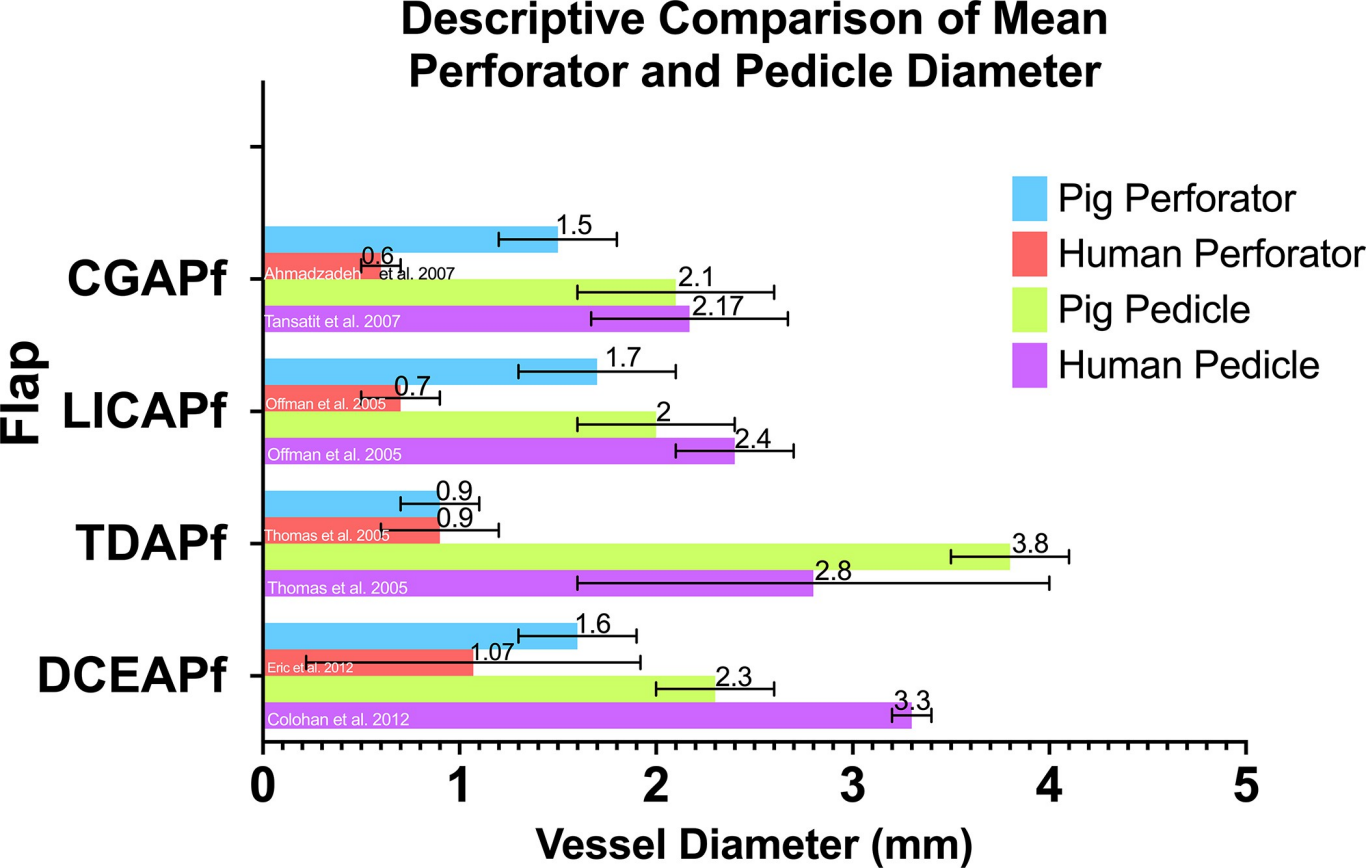
Descriptive comparison of perforator and pedicle diameter for the pig models and human counterpart for each flap type. DCAPf has no human correspondent.

**Fig 6:**
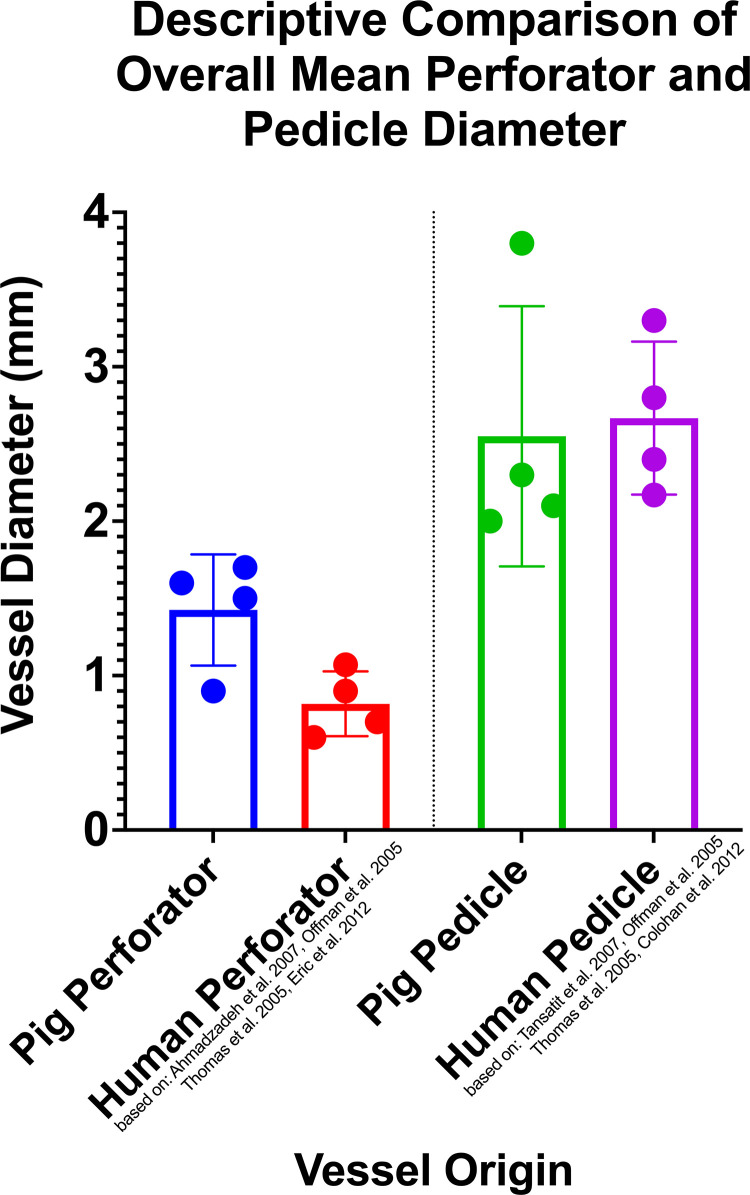
Descriptive overall comparison between the average diameters for all four flap types in pigs and humans. DCAPf has no human correspondent.

Perforator and pedicles of each four swine flaps match closely their human counterpart vessel in terms of diameter (Fig 5). When looking at the overall diameters, both perforator vessels as well as pedicles represent a close match for the human corresponding vessels (Fig 6).

### Flap harvesting times

**Fig 7:**
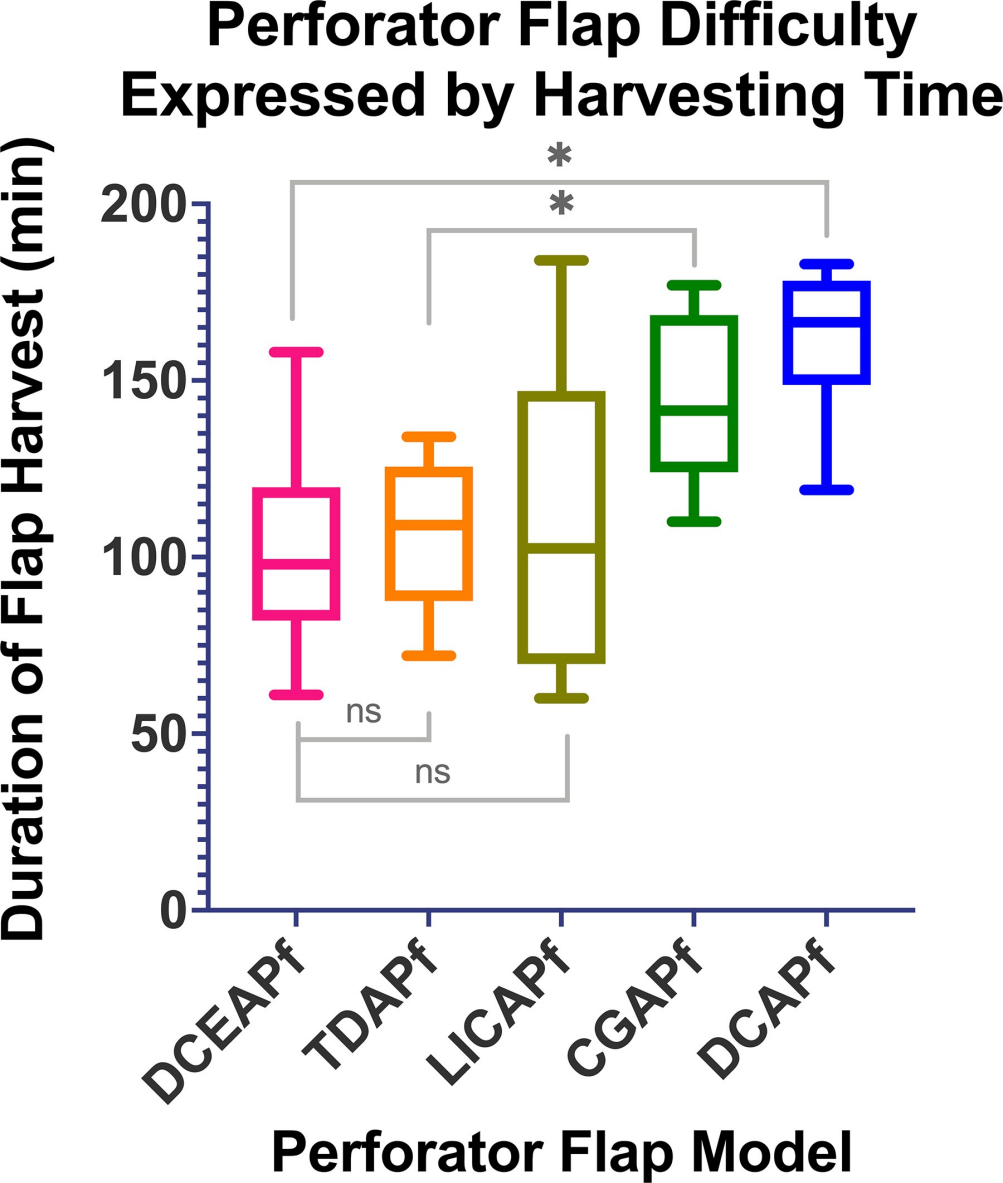
Difficulty of harvesting each flap. Was assessed based on the mean time required for each perforator flap type to be harvested. ns marks no significant statistical difference; * marks a significant statistical difference, with P<0.05.

**Fig 8:**
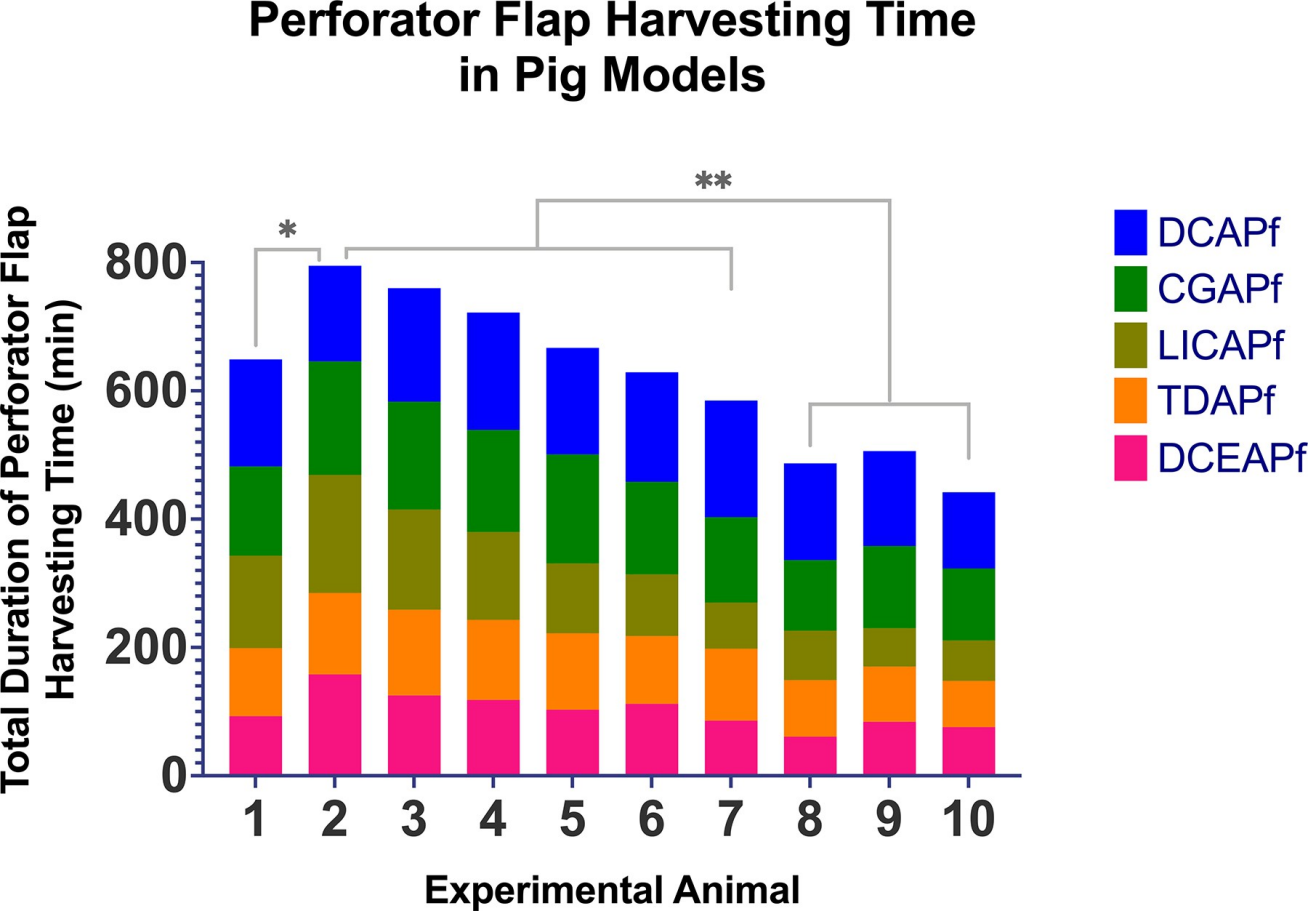
Total duration of perforator flap harvesting time. Was calculated as the sum of all individual perforator flap durations required for each of the five flaps performed in each of the 10 experimental animals. ns marks no significant statistical difference; * marks a significant statistical difference, with P<0.05; ** marks a very significant statistical difference, with P<0.01.

Average flap harvest duration varied (Fig 7), with DCAPf requiring the longest harvesting time due to both musculo- and septocutanous perforators present in the flap. The combined flap harvesting duration for all five flaps in each pig (Fig 8) decreased highly significantly between 2^nd^-7^th^ group and 8^th^-10^th^ experimental animal group.

**Fig 9:**
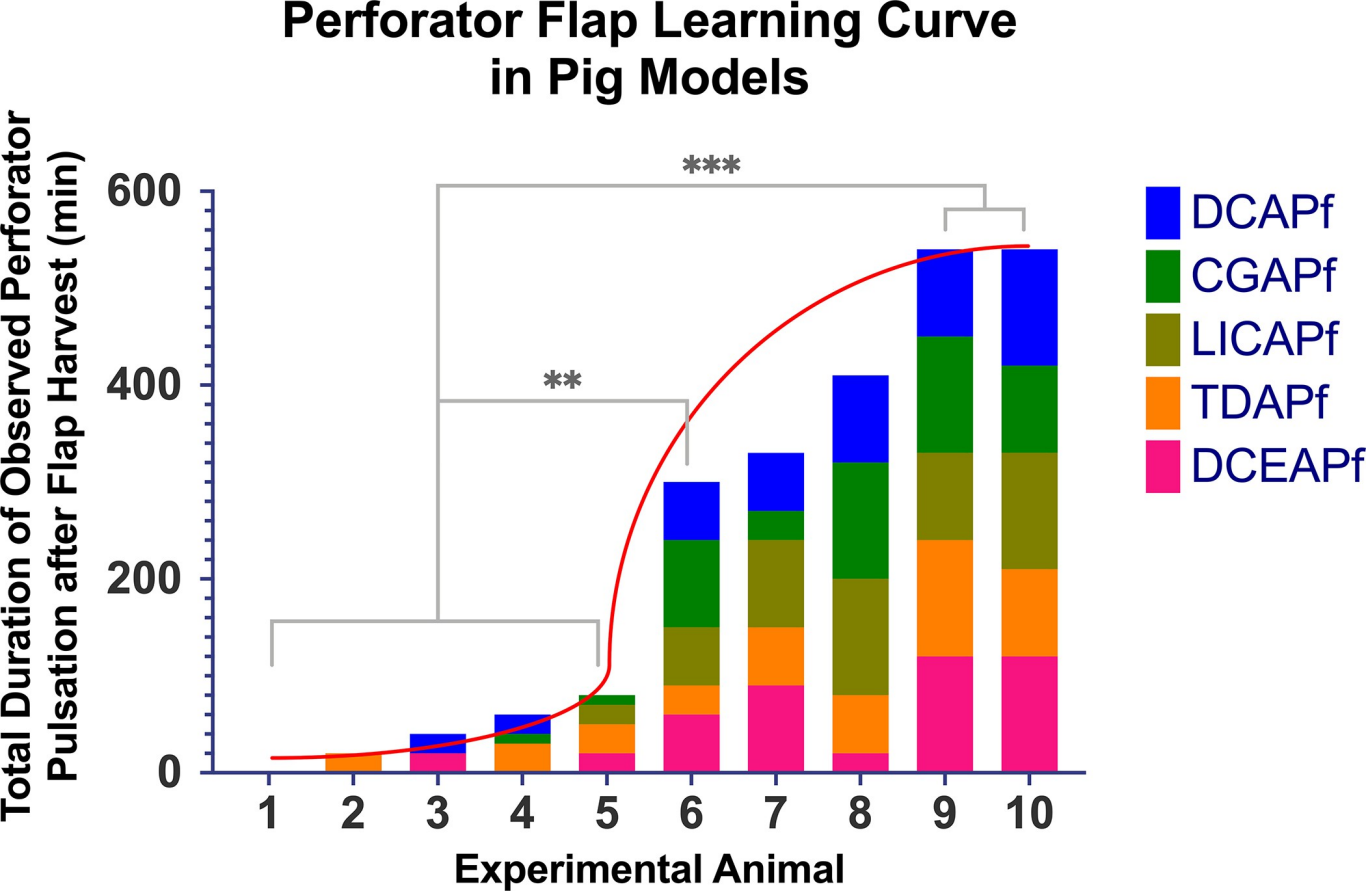
Plotting of the perforator flap learning curve (red) in pig models by aggregating the total duration of observed perforator pulsation after dissection of the perforator flap. A longer observed pulsation implies a better surgical outcome. ns marks no significant statistical difference; * marks a significant statistical difference, with P<0.05; ** marks a very significant statistical difference, with P<0.01; *** marks a highly significant statistical difference, with P<0.001.

Measuring how long a perforator remained pulsatile after flap harvesting allowed for objective quantification of the learning curve associated with harvesting perforator flaps (Fig 9). Post-operative perforator pulsation time revealed a steep learning curve, with no or short-term pulsatile perforators in the first five pigs, followed by a 275% increase in total perforator pulsation time between 5^th^ and 6^th^ experimental animal, indicating that even 6 one-day sessions of experimental surgery on pigs can lead to a dramatic increase in perforator flap dissection quality.

## Discussions

The American Society of Plastic Surgery highlights in its 2020 Statistics Report [26] a 75% increase in breast reconstruction procedures from 2000 to 2020, but the DIEP flap was used in only 9% of reconstructions [27]. This is because successful perforator flap surgery demands precise microsurgical skills, attainable only through extensive training featuring a steep learning curve [28]. Concerns regarding patient safety and costs of prolonged interventions limit direct operating room surgical training,[5] since muscle perforator flaps require a more delicate, time-consuming approach to dissection and tissue handling,[29] compared to free flap harvesting. Caution is required during intramuscular dissection, implying a steep learning curve which can be minimized by formal training courses, cadaver dissections, expert assistance and simulations.[4]

Surgical training which uses *in-vivo* animal models has proven as an invaluable educational tool in the last two decades,[6] being used extensively to supplement the shirking exposure to OR time during residency training programs, as shown by Bergmeister et al. This is especially true in light of the imposed reductions in the weekly work load of surgical residents in Europe and US alike.[1]

Fortunately, these five new experimental perforator flap models allow surgeons, even with no previous experience in perforator dissection, to supplement their skillset in a safe, controlled experimental surgery environment.

### The pig as an ideal training model for perforator flap dissection

The optimal animal model for surgical training is one that closely replicates the clinical scenario and is readily available.[13] The animal model should be simple to set up, animal-friendly, and reproducible. Additionally, it should provide a means of determining if trainees have progressed after practicing on the model. The ideal model would contain not just the technical ability necessary to conduct the surgery, but also provide consistent feed-back which can be translated into problem resolution, similar to aviation industry simulations, which emphasizes problem resolution and scenario management.

A review by Loh et al. [6] clearly indicated that large animal models are extremely valued for surgical training and that the pig is extensively used throughout *in-vivo* surgical simulation scenarios. Bergmeister et al. [1] showed in their systematic review that out of 91 publications describing *in-vivo* animal models used in surgical training, 70% used a porcine animal model and 24% involved trauma & reconstructive surgery surgical models. Compared to this, only 10% of these publications described murine surgical models, since perforator flaps in rats [9,10] exhibit perforator diameters of 0.2–0.3 mm [11] and pedicle diameters of 0.4–0.6 mm, [30] requiring thus supramicrosurgical techniques and instruments.

Cadavers are often considered to provide a suitable simulation of the surgical procedure. There are clear benefits to using a cadaveric model over live animals since they can be readily replicated and are often less expensive. Additionally, it eliminates the need for ethical clearance prior to using an animal practice model. Nevertheless, freshly frozen cadaver flap models, even with latex-injected colored vessels, represent a good opportunity to learn the anatomical landmarks but fail to provide critical surgical feedback as bleeding tissues do, thus rendering them unsuitable for perforator flap training.

Additionally, as a competency-based evaluation, animal models may be utilized to evaluate a trainee’s progress during yearly surgical reviews. Learning new methods may be accomplished during a period of rigorous instruction, such as a perforator flap harvesting courses [31]. While animal model simulation will never be a substitute for clinical training, it may be used to refine a trainee’s expertise, enhancing their confidence in the clinical situation and decreasing the learning curve. Operating room conditions can be simulated only within in-vivo large animal models, with pigs accurately simulating the human vascular anatomy [32] and pathophysiology.[33]

Our findings indicate that pigs of Large White breed, weighing 29–36 kg, can be consistently used to harvest the described perforator flaps which accurately simulate the perforator and pedicle diameters of the four corresponding human perforator flaps, as shown in Table 1 and in Figs 5 and 6.

### Team training approach

The 3Rs principles [34] imposed modifications to the perforator models by dividing the midline flaps DCEAPf and DCAPf in two half-flaps, allowing simultaneous surgical training and a decrease in required experimental animals. If required, the entire area of these flaps can be harvested as a single flap, based on one or multiple perforators.

These perforator flaps can be harvested simultaneous on both sides of the pig by two surgeons. However, we encourage the operator-assistant team training approach, with two surgeons exchanging the operator role, each one having allotted a hemi-body of the pig. This allows for greater precision and attention to detail, having the assistant as a constant monitoring factor. Bergmeister et al. [1] highlight in their systematic review that the team training approach with large animal models is used throughout the various surgical specialties to simulate a specific situation, such as intraabdominal bleeding, and to optimize teamwork in critical situations.

### Key surgical techniques during experimental perforator flap harvesting

Doppler sonography suffices for flap planning, [35] but color Doppler duplex ultrasound can be used if desired. Correct technique and previous experience are recommended before using Doppler ultrasound to identify perforator vessels in the pig models. Failing to do so will result in false-negative and false-positive findings. Using the described landmarks in pigs, failure to identify at least one perforator during surgical approach of the flap is highly unlikely.

During surgery, usage of soft bipolar coagulation (VIO 300D, Erbe, Germany) with non-stick micro-bipolar forceps allows for precise dissection of the perforator flaps in pigs, with minimal bleeding and a clean, blood-free surgical field. Needle-tip monopolar dissection with low current intensity is used away from the main perforator and pedicle.

The depth of the operating field varies from subcutaneous (LICAPf) to 6–8 cm (DCAPf), allowing for different training situations. All pig perforator models allow for optional microsurgical anastomosis after flap harvesting.

Harvesting time increased significantly between the 1^st^ and 2^nd^ pig, after which it steadily decreased with each new experiment.

Most flap harvesting failures resulted from rushing the dissection with no or short perforator pulsation in flaps harvested on the 1^st^-5^th^ pig (Fig 9). An increase of 22.4% in total harvesting time was observed between the first and second experimental animal. Due to the poor outcome in flap survival for the first experimental animal, as measured in terms of duration of perforator pulsation, the surgical approach for the second experimental animal was done with increased caution and attention to the intramuscular dissection, thus leading to a longer total harvesting time. Starting with the second experimental animal, with each consecutive pig, the total harvesting time decreased as expected when observing repetitive procedures. As such, total decrease of 44.4% between second and last experiment can be noticed.

A brief description of most common mistakes, consequences and gestures which can be trained using the pig perforator flaps, in order to overcome these mistakes, can be found in Table 2.

**Table 2:** Skills to be trained using the pig perforator flap models.

*Mistakes during perforator flap dissection*	*Consequence*	*Gestures and attitudes to be learned while dissecting perforator flaps*
Rushing the perforator and pedicle dissection. Coagulation of perforator and pedicle branches using bipolar.	Irreversible damage to perforator or pedicle	“Cut only what you see!” Slowing down during perforator and pedicle dissection. Usage of micro titanium hemostatic clips for transection of perforator and pedicle branches.
Poor intramuscular perforator or pedicle dissection. Incorrect surgeon’s position.	Accidental damage to perforator or pedicle. Failure to notice sudden changes in perforator course. Inability to control fine movements.	Perforator dissection with muscle cuff. De-roofing of perforator with bipolar coagulation. Following and anticipating the perforator’s course in the muscle fibers. Sitting position, elbow and wrist support, firm hand support on body region while dissecting.
Dissecting too close to the perforator or pedicle.	Inducing perforator and/or pedicle spasm	“No-touch approach” Avoiding dissection of perforator with scissors opening perpendicularly to the perforator
Dissection of flap island with scissors. Dissection of perforator and pedicle with micro-scissors.	Long perforator flap harvesting times. Uncontrolled bleeding from perforator and pedicle side branches.	Soft tissue dissection with needle-tip monopolar on low-intensity current, away from perforator and pedicle. De-roofing of perforator with bipolar coagulation.
Failure to collaborate with the anesthesiologist	Low blood pressure in experimental animal. Accidental cutting of spastic perforator.	Assuring an elevated blood pressure and body temperature throughout surgery. Constant blood pressure monitoring by anesthesiologist while dissecting the perforator.
No supervision of the assistant.	Traction on the perforator and/or pedicle. Desiccation of the perforator and/or pedicle. Operating field full of blood.	Accurate control by surgeon of tension applied to both perforator and pedicle. Constant irrigation of perforator and pedicle. Minimizing bleeding by constant soft bipolar coagulation of dissected tissue.

### Cost-analysis of experimental models

Since each experimental animal provides 10 perforator flaps (5 on each side), these 5 new pig experimental perforator flaps can be considered cost-efficient. This is the case even when considering the costs involved for purchasing the experimental animal, inhalatory anesthesia or TIVA and incineration as medical waste after euthanasia.

Bergmeister et al. [1] performed a cost analysis of large animal surgical simulations in pigs, calculated as a one-day training event using a single pig. They have shown a cost ranging 320–634€ (362–718$) for animal acquisition, transport and facilities. Anesthesia and perioperative care, including equipment, staff and medication, averaged 415€ (470$) per one training event. Since perforator flap harvesting does not necessitate dedicated consumables and single use equipment, other than the microsurgical sutures and microvascular hemoclips, the total cost for a one-day training event ultimately comes down to the institutional costs involved, covering administrative costs and operating room rental, which alone averaged 1110€ (1258$). Compared to this, our total costs for a one-day training event, involving two trainees and one pig are averaging 593€ (672$). Nevertheless, an animal surgical research facility, capable of providing adequate animal care and an operating theater with general anesthesia, is required in order to safely perform these training models.

## Conclusions

These five pig perforator flap models represent an excellent training tool for reconstructive surgeons aiming to develop their skills outside the surgical operating room. Given their close match with corresponding human flaps, in terms of vessel caliber and length, we consider these experimental models best suited for perforator flap training worldwide.

Presented at the 59th Annual Plastic Surgery Research Council (PSRC) Meeting, New York, USA.

## Supporting information

S1 Video 3D-VRT pig vascular network model and perforator flaps localization. Three-dimensional volume-rendering (3D-VRT) reconstruction of an entire experimental animal. Using a 64-detector scanner we obtained a high resolution of the skin surface and the underlying vascular network, which revealed five perforasomes of interest with correspondents in human flap anatomy. The location of the five designated flaps can be seen in the last sequence of rotation.
(MP4)

S2 Video 3D-VRT of DCEAPf flap planning and landmarks. Three-dimensional volume-rendering (3D-VRT) reconstruction of the abdominal surface of the pig, with underlying vascular architecture exposed. Deep cranial epigastric artery perforator flap (DCEAPf) planning is shown with specific landmarks.
(MP4)

S3 Video 3D-VRT pig hemithorax model, depicting TDAPf positioning. Three-dimensional volume-rendering (3D-VRT) reconstruction of the hemithorax of the pig, with underlying vascular architecture exposed. Thoracodorsal artery perforator flap (TDAPf) planning is shown with specific landmarks.
(MP4)

S4 Video 3D-VRT pig abdomen model, depicting LICAPf planning. Three-dimensional volume-rendering (3D-VRT) reconstruction of the hemithorax of the pig, with underlying vascular architecture exposed. Lateral intercostal artery perforator flap (LICAPf) planning is shown with specific landmarks.
(MP4)

S5 Video 3D-VRT pig buttock model, depicting CGAPf planning. Three-dimensional volume-rendering (3D-VRT) reconstruction of the pig buttock, with underlying vascular architecture exposed. Cranial gluteal artery perforator flap (CGAPf) planning is shown with specific landmarks.
(MP4)

S6 Video 3D-VRT pig dorsal cervical model, depicting DCAPf planning. Three-dimensional volume-rendering (3D-VRT) reconstruction of the dorsal cervical region of the pig, with underlying vascular architecture exposed. Deep cervical artery perforator flap (DCAPf) planning is shown with specific landmarks.
(MP4)

S1 File (DOCX)
