# Supplementary material for: Experimental swine models for perforator flap dissection in reconstructive microsurgery
Source: PLoS One. 2022 Apr 11;17(4):e0266873. doi: 10.1371/journal.pone.0266873 (PMC9000060; doi:10.1371/journal.pone.0266873)

**Supporting Information**

**Statistical analysis of data**

1. **Pig perforator diameter normality test**

DESCRIPTIVES VARIABLES=DCEAPf TDAPf LICAPf CGAPf DCAPf

/STATISTICS=MEAN STDDEV MIN MAX KURTOSIS SKEWNESS.

**Conclusion**: The Skewness and Kurtosis are between -1.96 and 1.96 (except for CGAPf Kurtosis), both Shapiro-Wilk and Kolmogorov-Smirnov tests have p-values >0.05, and the normal Q-Q plot shows a relatively normal distribution. Therefore, we conclude that, even if the sample size is small (n=10), the data is normally distributed.

**Descriptives**

| **Notes** | | |
| --- | --- | --- |
| Output Created | | 02-SEP-2021 22:12:55 |
| Comments | |  |
| Input | Data | \pig perforator diameter.sav |
|  | Active Dataset | DataSet1 |
|  | Filter | <none> |
|  | Weight | <none> |
|  | Split File | <none> |
|  | N of Rows in Working Data File | 10 |
| Missing Value Handling | Definition of Missing | User defined missing values are treated as missing. |
|  | Cases Used | All non-missing data are used. |
| Syntax | | DESCRIPTIVES VARIABLES=DCEAPf TDAPf LICAPf CGAPf DCAPf  /STATISTICS=MEAN STDDEV MIN MAX KURTOSIS SKEWNESS. |
| Resources | Processor Time | 00:00:00.02 |
|  | Elapsed Time | 00:00:00.03 |

| **Descriptive Statistics** | | | | | | | | | |
| --- | --- | --- | --- | --- | --- | --- | --- | --- | --- |
|  | N | Minimum | Maximum | Mean | Std. Deviation | Skewness | | Kurtosis | |
|  | Statistic | Statistic | Statistic | Statistic | Statistic | Statistic | Std. Error | Statistic | Std. Error |
| DCEAPf | 10 | 1.0 | 2.0 | 1.590 | .2846 | -.778 | .687 | 1.186 | 1.334 |
| TDAPf | 10 | .6 | 1.3 | .890 | .2025 | .369 | .687 | 1.213 | 1.334 |
| LICAPf | 10 | .3 | 1.3 | .750 | .3472 | .328 | .687 | -1.490 | 1.334 |
| CGAPf | 10 | 1.0 | 2.1 | 1.460 | .2914 | .833 | .687 | 2.281 | 1.334 |
| DCAPf | 10 | 1.1 | 2.0 | 1.540 | .2951 | .093 | .687 | -1.162 | 1.334 |
| Valid N (listwise) | 10 |  |  |  |  |  |  |  |  |

EXAMINE VARIABLES=DCEAPf TDAPf LICAPf CGAPf DCAPf

/PLOT BOXPLOT HISTOGRAM NPPLOT

/COMPARE GROUPS

/STATISTICS DESCRIPTIVES

/CINTERVAL 95

/MISSING LISTWISE

/NOTOTAL.

**Explore**

| **Notes** | | |
| --- | --- | --- |
| Output Created | | 02-SEP-2021 22:13:32 |
| Comments | |  |
| Input | Data | pig perforator diameter.sav |
|  | Active Dataset | DataSet1 |
|  | Filter | <none> |
|  | Weight | <none> |
|  | Split File | <none> |
|  | N of Rows in Working Data File | 10 |
| Missing Value Handling | Definition of Missing | User-defined missing values for dependent variables are treated as missing. |
|  | Cases Used | Statistics are based on cases with no missing values for any dependent variable or factor used. |
| Syntax | | EXAMINE VARIABLES=DCEAPf TDAPf LICAPf CGAPf DCAPf  /PLOT BOXPLOT HISTOGRAM NPPLOT  /COMPARE GROUPS  /STATISTICS DESCRIPTIVES  /CINTERVAL 95  /MISSING LISTWISE  /NOTOTAL. |
| Resources | Processor Time | 00:00:04.89 |
|  | Elapsed Time | 00:00:02.97 |

| **Case Processing Summary** | | | | | | |
| --- | --- | --- | --- | --- | --- | --- |
|  | Cases | | | | | |
|  | Valid | | Missing | | Total | |
|  | N | Percent | N | Percent | N | Percent |
| DCEAPf | 10 | 100.0% | 0 | 0.0% | 10 | 100.0% |
| TDAPf | 10 | 100.0% | 0 | 0.0% | 10 | 100.0% |
| LICAPf | 10 | 100.0% | 0 | 0.0% | 10 | 100.0% |
| CGAPf | 10 | 100.0% | 0 | 0.0% | 10 | 100.0% |
| DCAPf | 10 | 100.0% | 0 | 0.0% | 10 | 100.0% |

| **Descriptives** | | | | |
| --- | --- | --- | --- | --- |
|  | | | Statistic | Std. Error |
| DCEAPf | Mean | | 1.590 | .0900 |
|  | 95% Confidence Interval for Mean | Lower Bound | 1.386 |  |
|  |  | Upper Bound | 1.794 |  |
|  | 5% Trimmed Mean | | 1.600 |  |
|  | Median | | 1.600 |  |
|  | Variance | | .081 |  |
|  | Std. Deviation | | .2846 |  |
|  | Minimum | | 1.0 |  |
|  | Maximum | | 2.0 |  |
|  | Range | | 1.0 |  |
|  | Interquartile Range | | .3 |  |
|  | Skewness | | -.778 | .687 |
|  | Kurtosis | | 1.186 | 1.334 |
| TDAPf | Mean | | .890 | .0640 |
|  | 95% Confidence Interval for Mean | Lower Bound | .745 |  |
|  |  | Upper Bound | 1.035 |  |
|  | 5% Trimmed Mean | | .883 |  |
|  | Median | | .900 |  |
|  | Variance | | .041 |  |
|  | Std. Deviation | | .2025 |  |
|  | Minimum | | .6 |  |
|  | Maximum | | 1.3 |  |
|  | Range | | .7 |  |
|  | Interquartile Range | | .3 |  |
|  | Skewness | | .369 | .687 |
|  | Kurtosis | | 1.213 | 1.334 |
| LICAPf | Mean | | .750 | .1098 |
|  | 95% Confidence Interval for Mean | Lower Bound | .502 |  |
|  |  | Upper Bound | .998 |  |
|  | 5% Trimmed Mean | | .744 |  |
|  | Median | | .650 |  |
|  | Variance | | .121 |  |
|  | Std. Deviation | | .3472 |  |
|  | Minimum | | .3 |  |
|  | Maximum | | 1.3 |  |
|  | Range | | 1.0 |  |
|  | Interquartile Range | | .6 |  |
|  | Skewness | | .328 | .687 |
|  | Kurtosis | | -1.490 | 1.334 |
| CGAPf | Mean | | 1.460 | .0921 |
|  | 95% Confidence Interval for Mean | Lower Bound | 1.252 |  |
|  |  | Upper Bound | 1.668 |  |
|  | 5% Trimmed Mean | | 1.450 |  |
|  | Median | | 1.450 |  |
|  | Variance | | .085 |  |
|  | Std. Deviation | | .2914 |  |
|  | Minimum | | 1.0 |  |
|  | Maximum | | 2.1 |  |
|  | Range | | 1.1 |  |
|  | Interquartile Range | | .3 |  |
|  | Skewness | | .833 | .687 |
|  | Kurtosis | | 2.281 | 1.334 |
| DCAPf | Mean | | 1.540 | .0933 |
|  | 95% Confidence Interval for Mean | Lower Bound | 1.329 |  |
|  |  | Upper Bound | 1.751 |  |
|  | 5% Trimmed Mean | | 1.539 |  |
|  | Median | | 1.450 |  |
|  | Variance | | .087 |  |
|  | Std. Deviation | | .2951 |  |
|  | Minimum | | 1.1 |  |
|  | Maximum | | 2.0 |  |
|  | Range | | .9 |  |
|  | Interquartile Range | | .5 |  |
|  | Skewness | | .093 | .687 |
|  | Kurtosis | | -1.162 | 1.334 |

| **Tests of Normality** | | | | | | |
| --- | --- | --- | --- | --- | --- | --- |
|  | Kolmogorov-Smirnov^a^ | | | Shapiro-Wilk | | |
|  | Statistic | df | Sig. | Statistic | df | Sig. |
| DCEAPf | .214 | 10 | .200^*^ | .936 | 10 | .514 |
| TDAPf | .220 | 10 | .188 | .899 | 10 | .215 |
| LICAPf | .167 | 10 | .200^*^ | .918 | 10 | .340 |
| CGAPf | .215 | 10 | .200^*^ | .931 | 10 | .455 |
| DCAPf | .211 | 10 | .200^*^ | .927 | 10 | .421 |
| *. This is a lower bound of the true significance. | | | | | | |
| a. Lilliefors Significance Correction | | | | | | |

**DCEAPf**


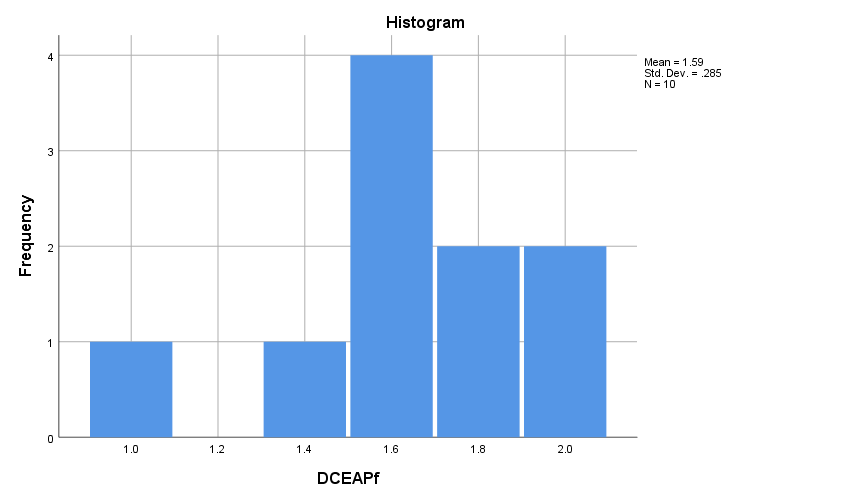

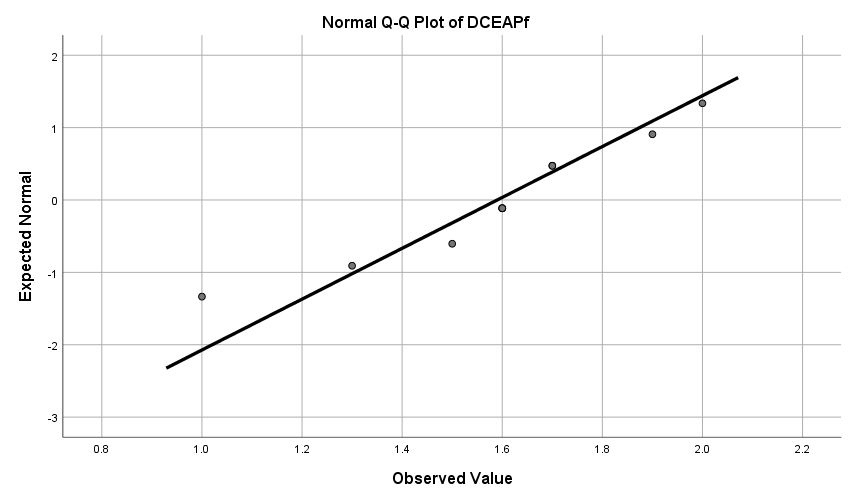


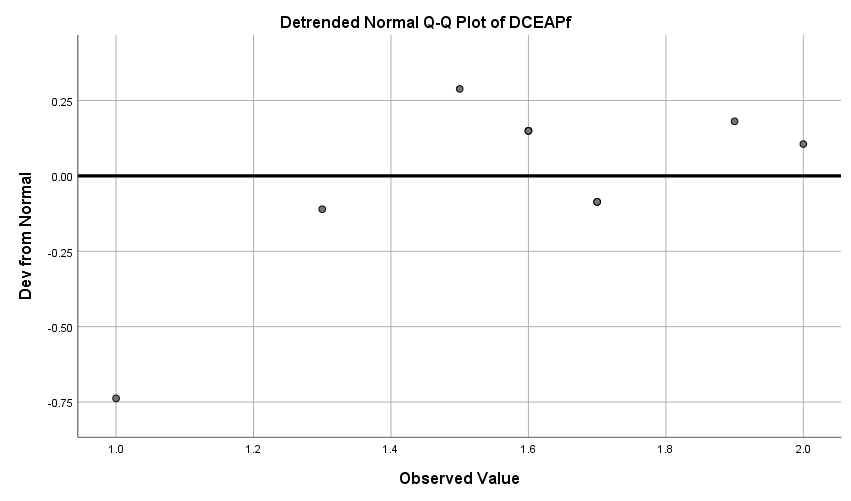

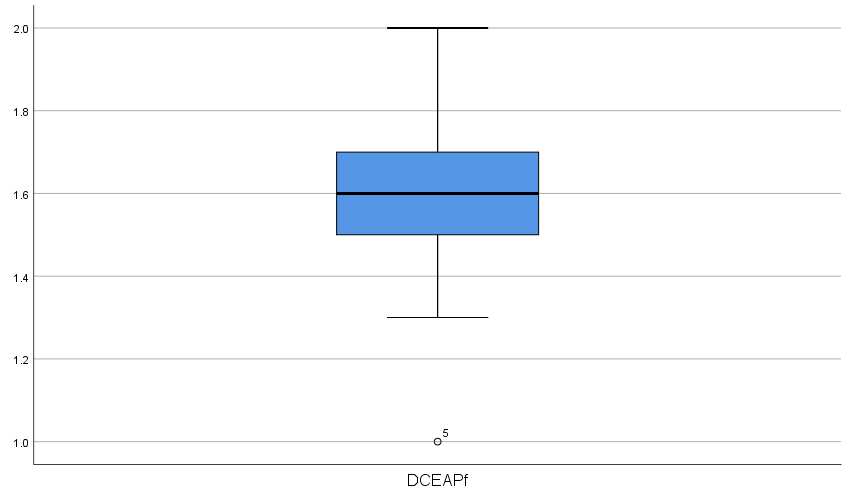


**TDAPf**


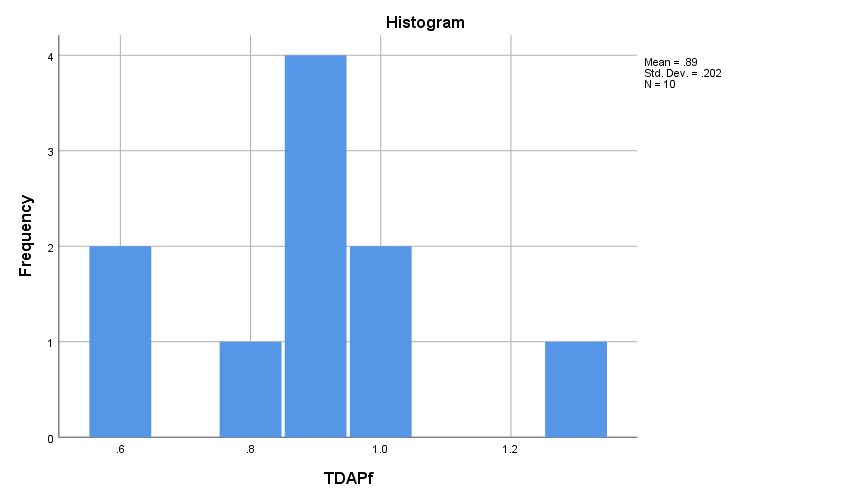

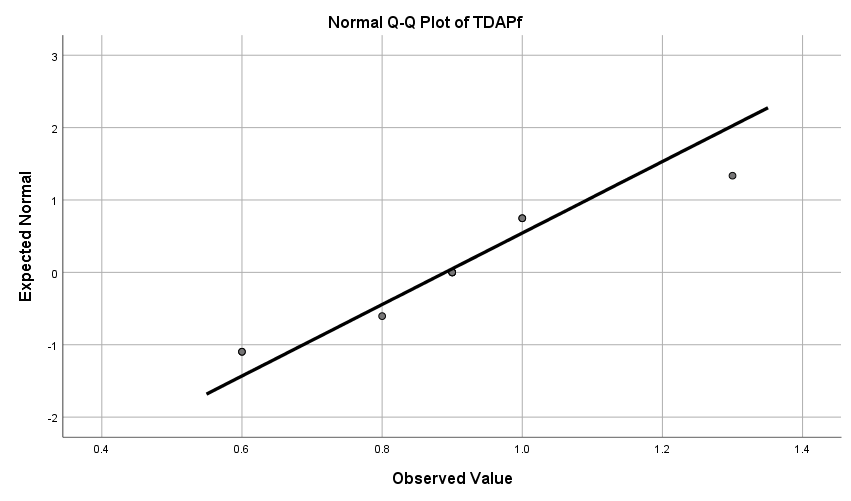


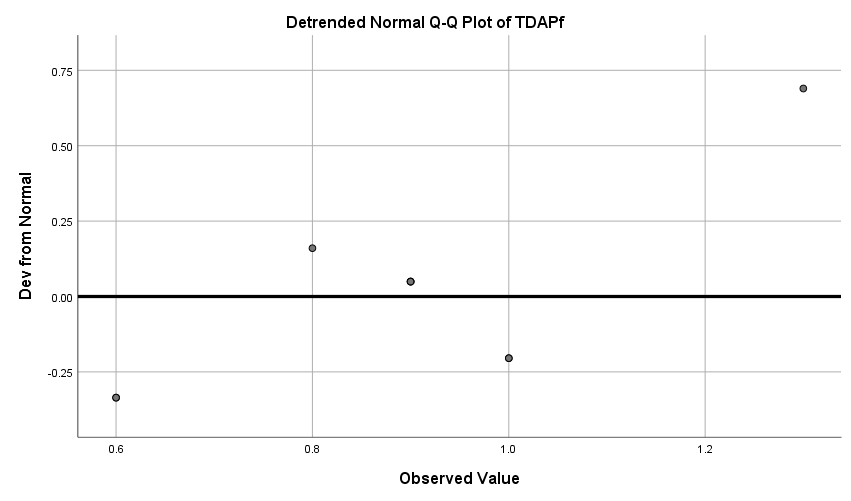

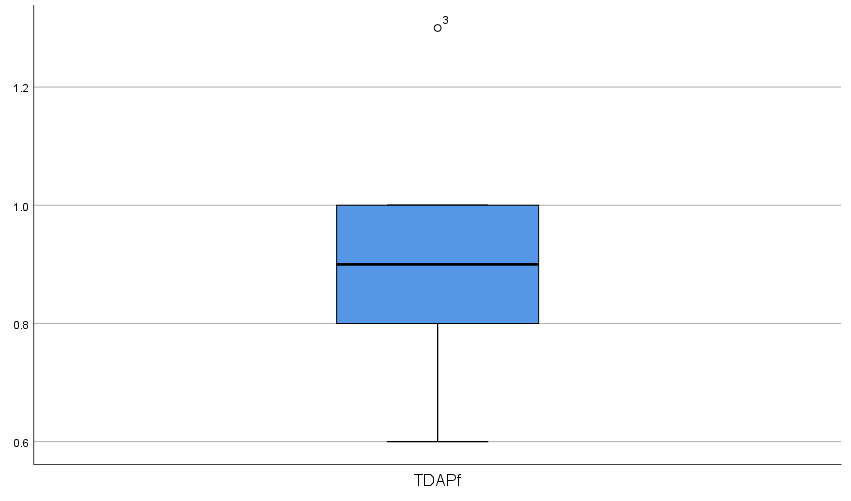


**LICAPf**


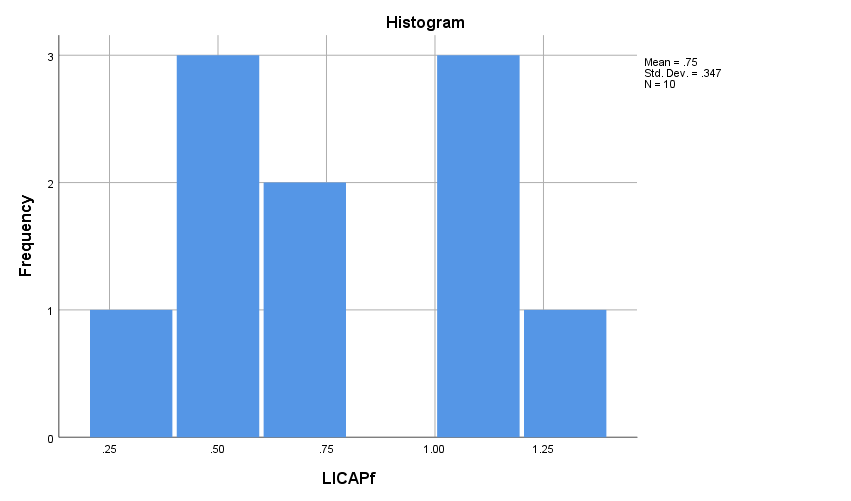

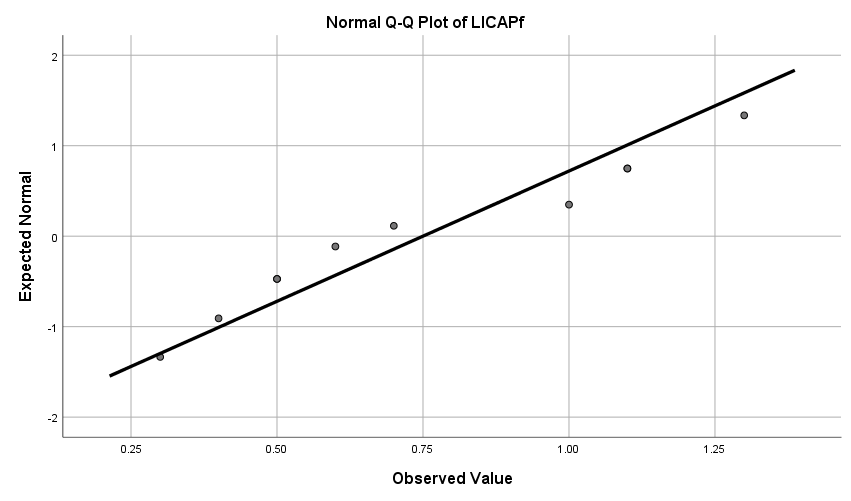


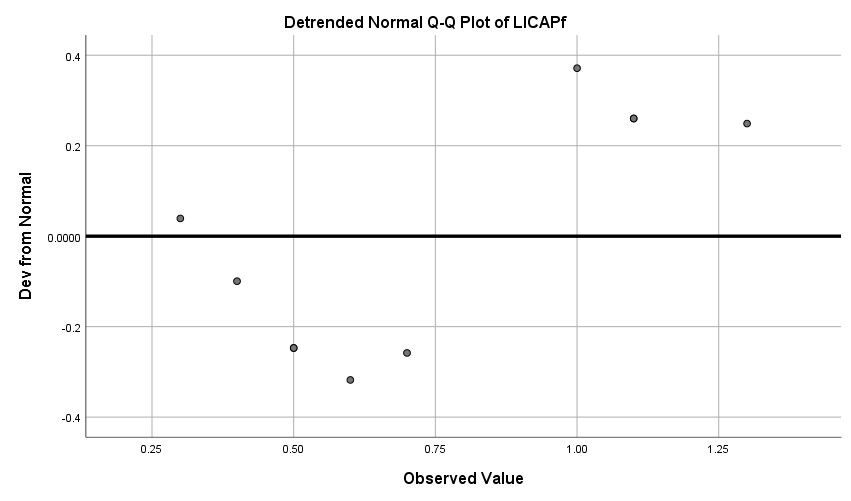

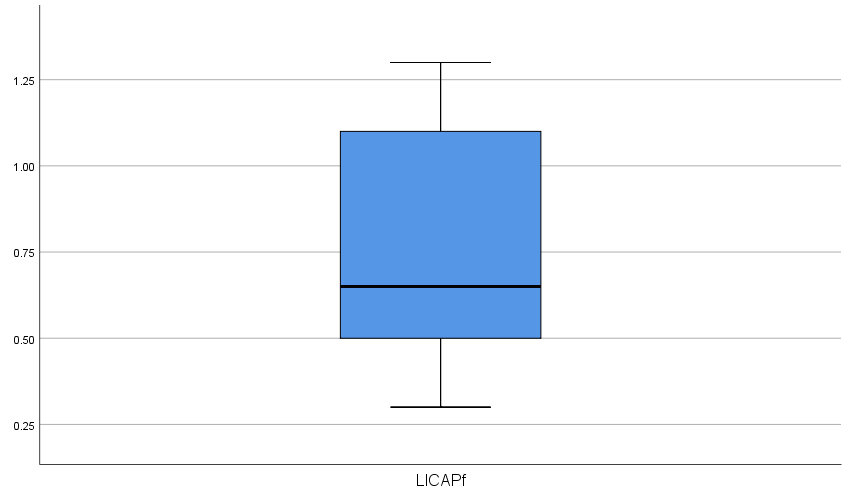


**CGAPf**


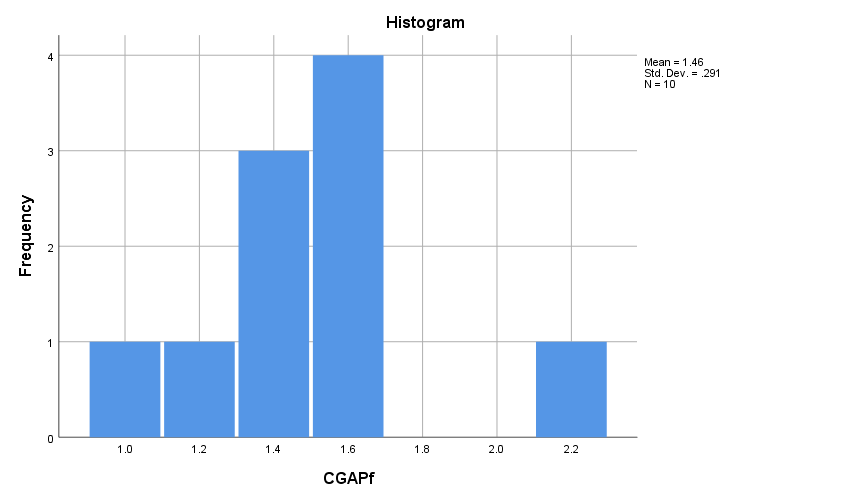

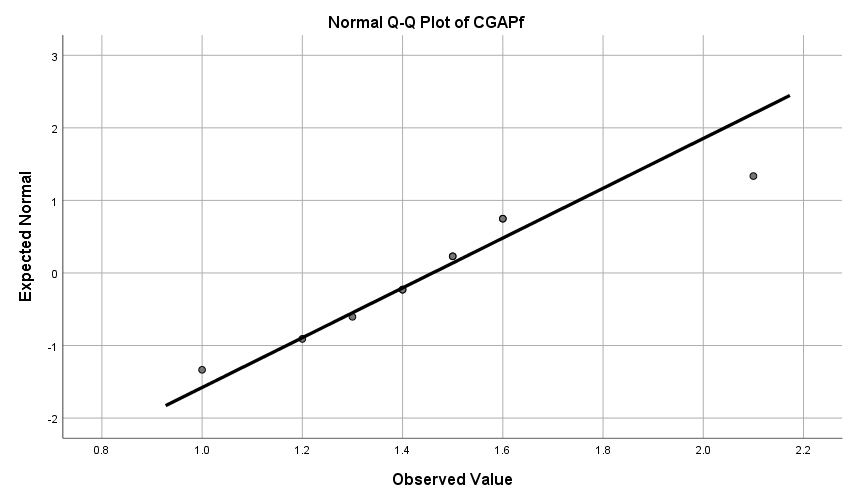


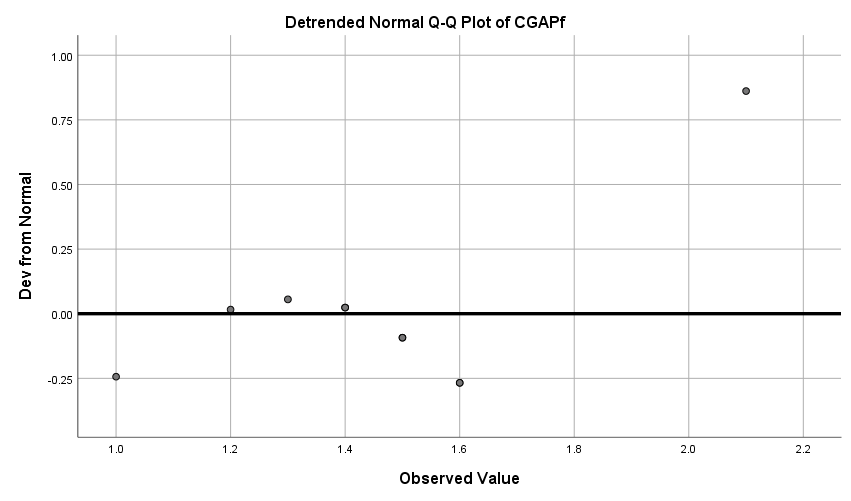

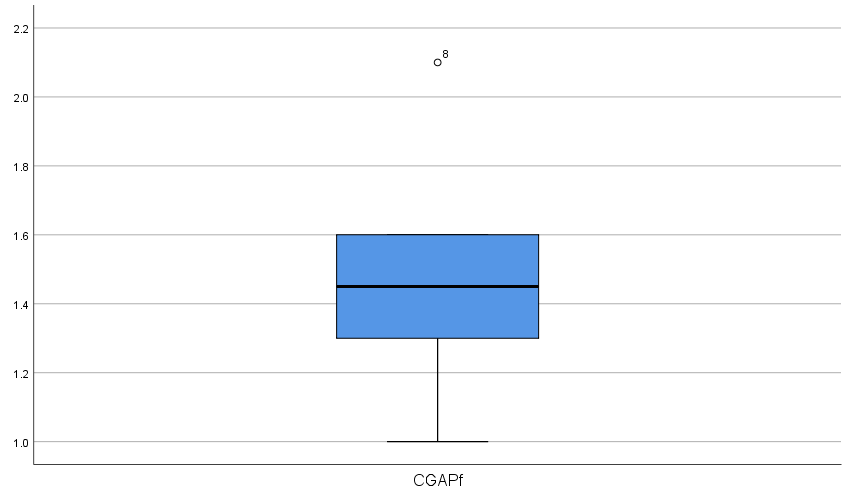


**DCAPf**


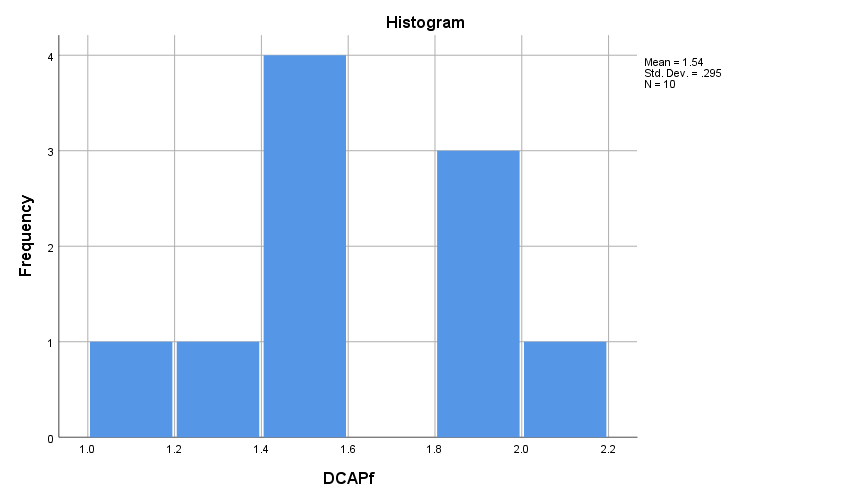

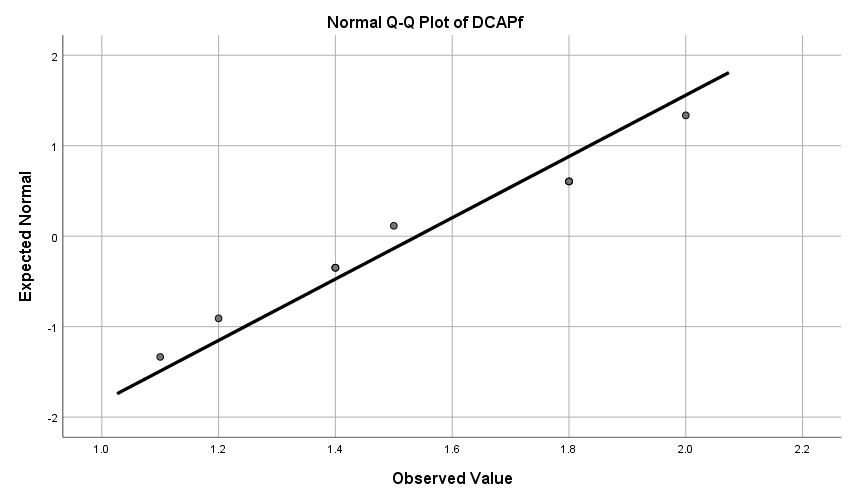


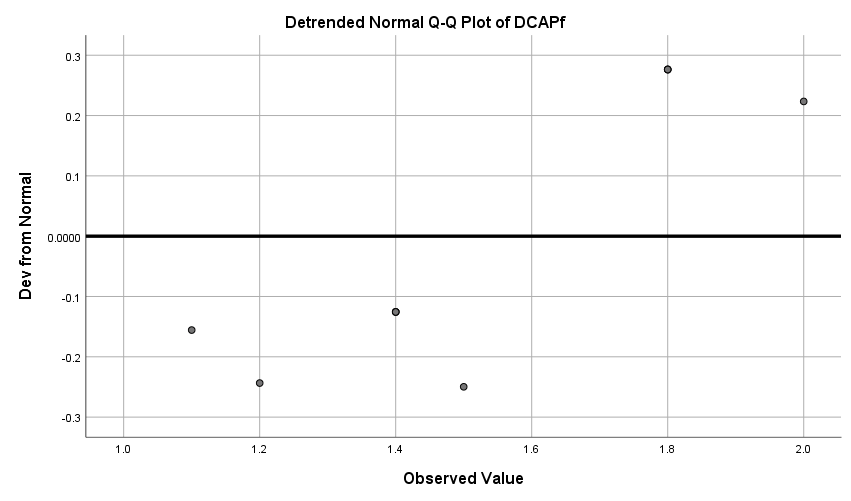

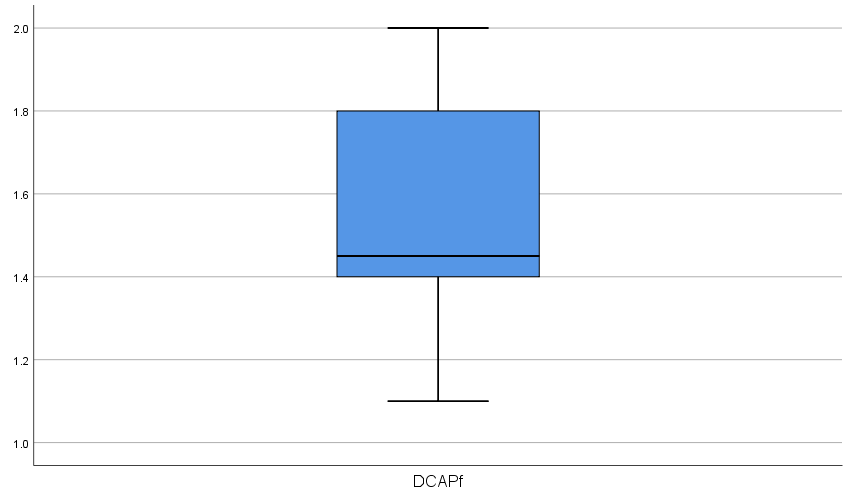


1. **Pig pedicle diameter normality test**

DESCRIPTIVES VARIABLES=DCEAPf TDAPf LICAPf CGAPf DCAPf

/STATISTICS=MEAN STDDEV MIN MAX KURTOSIS SKEWNESS.

**Conclusion**: The Skewness and Kurtosis are between -1.96 and 1.96, both Shapiro-Wilk and Kolmogorov-Smirnov tests have p-values >0.05, and the normal Q-Q plot shows a relatively normal distribution. Therefore, we conclude that, even if the sample size is small (n=10), the data is normally distributed.

**Descriptives**

| **Notes** | | |
| --- | --- | --- |
| Output Created | | 02-SEP-2021 22:19:13 |
| Comments | |  |
| Input | Data | pig pedicle diameter.sav |
|  | Active Dataset | DataSet1 |
|  | Filter | <none> |
|  | Weight | <none> |
|  | Split File | <none> |
|  | N of Rows in Working Data File | 10 |
| Missing Value Handling | Definition of Missing | User defined missing values are treated as missing. |
|  | Cases Used | All non-missing data are used. |
| Syntax | | DESCRIPTIVES VARIABLES=DCEAPf TDAPf LICAPf CGAPf DCAPf  /STATISTICS=MEAN STDDEV MIN MAX KURTOSIS SKEWNESS. |
| Resources | Processor Time | 00:00:00.02 |
|  | Elapsed Time | 00:00:00.08 |

| **Descriptive Statistics** | | | | | | | | | |
| --- | --- | --- | --- | --- | --- | --- | --- | --- | --- |
|  | N | Minimum | Maximum | Mean | Std. Deviation | Skewness | | Kurtosis | |
|  | Statistic | Statistic | Statistic | Statistic | Statistic | Statistic | Std. Error | Statistic | Std. Error |
| DCEAPf | 10 | 1.7 | 2.8 | 2.300 | .3266 | -.215 | .687 | .057 | 1.334 |
| TDAPf | 10 | 3.4 | 4.3 | 3.810 | .3035 | .001 | .687 | -1.052 | 1.334 |
| LICAPf | 10 | 1.5 | 2.6 | 2.030 | .4218 | -.251 | .687 | -1.569 | 1.334 |
| CGAPf | 10 | 1.0 | 2.9 | 2.070 | .5870 | -.655 | .687 | -.224 | 1.334 |
| DCAPf | 10 | 3.3 | 4.5 | 3.790 | .4122 | .648 | .687 | -.529 | 1.334 |
| Valid N (listwise) | 10 |  |  |  |  |  |  |  |  |

EXAMINE VARIABLES=DCEAPf TDAPf LICAPf CGAPf DCAPf

/PLOT BOXPLOT HISTOGRAM NPPLOT

/COMPARE GROUPS

/STATISTICS DESCRIPTIVES

/CINTERVAL 95

/MISSING LISTWISE

/NOTOTAL.

**Explore**

| **Notes** | | |
| --- | --- | --- |
| Output Created | | 02-SEP-2021 22:19:29 |
| Comments | |  |
| Input | Data | \pig pedicle diameter.sav |
|  | Active Dataset | DataSet1 |
|  | Filter | <none> |
|  | Weight | <none> |
|  | Split File | <none> |
|  | N of Rows in Working Data File | 10 |
| Missing Value Handling | Definition of Missing | User-defined missing values for dependent variables are treated as missing. |
|  | Cases Used | Statistics are based on cases with no missing values for any dependent variable or factor used. |
| Syntax | | EXAMINE VARIABLES=DCEAPf TDAPf LICAPf CGAPf DCAPf  /PLOT BOXPLOT HISTOGRAM NPPLOT  /COMPARE GROUPS  /STATISTICS DESCRIPTIVES  /CINTERVAL 95  /MISSING LISTWISE  /NOTOTAL. |
| Resources | Processor Time | 00:00:03.27 |
|  | Elapsed Time | 00:00:02.66 |

| **Case Processing Summary** | | | | | | |
| --- | --- | --- | --- | --- | --- | --- |
|  | Cases | | | | | |
|  | Valid | | Missing | | Total | |
|  | N | Percent | N | Percent | N | Percent |
| DCEAPf | 10 | 100.0% | 0 | 0.0% | 10 | 100.0% |
| TDAPf | 10 | 100.0% | 0 | 0.0% | 10 | 100.0% |
| LICAPf | 10 | 100.0% | 0 | 0.0% | 10 | 100.0% |
| CGAPf | 10 | 100.0% | 0 | 0.0% | 10 | 100.0% |
| DCAPf | 10 | 100.0% | 0 | 0.0% | 10 | 100.0% |

| **Descriptives** | | | | |
| --- | --- | --- | --- | --- |
|  | | | Statistic | Std. Error |
| DCEAPf | Mean | | 2.300 | .1033 |
|  | 95% Confidence Interval for Mean | Lower Bound | 2.066 |  |
|  |  | Upper Bound | 2.534 |  |
|  | 5% Trimmed Mean | | 2.306 |  |
|  | Median | | 2.300 |  |
|  | Variance | | .107 |  |
|  | Std. Deviation | | .3266 |  |
|  | Minimum | | 1.7 |  |
|  | Maximum | | 2.8 |  |
|  | Range | | 1.1 |  |
|  | Interquartile Range | | .5 |  |
|  | Skewness | | -.215 | .687 |
|  | Kurtosis | | .057 | 1.334 |
| TDAPf | Mean | | 3.810 | .0960 |
|  | 95% Confidence Interval for Mean | Lower Bound | 3.593 |  |
|  |  | Upper Bound | 4.027 |  |
|  | 5% Trimmed Mean | | 3.806 |  |
|  | Median | | 3.850 |  |
|  | Variance | | .092 |  |
|  | Std. Deviation | | .3035 |  |
|  | Minimum | | 3.4 |  |
|  | Maximum | | 4.3 |  |
|  | Range | | .9 |  |
|  | Interquartile Range | | .5 |  |
|  | Skewness | | .001 | .687 |
|  | Kurtosis | | -1.052 | 1.334 |
| LICAPf | Mean | | 2.030 | .1334 |
|  | 95% Confidence Interval for Mean | Lower Bound | 1.728 |  |
|  |  | Upper Bound | 2.332 |  |
|  | 5% Trimmed Mean | | 2.028 |  |
|  | Median | | 2.200 |  |
|  | Variance | | .178 |  |
|  | Std. Deviation | | .4218 |  |
|  | Minimum | | 1.5 |  |
|  | Maximum | | 2.6 |  |
|  | Range | | 1.1 |  |
|  | Interquartile Range | | .8 |  |
|  | Skewness | | -.251 | .687 |
|  | Kurtosis | | -1.569 | 1.334 |
| CGAPf | Mean | | 2.070 | .1856 |
|  | 95% Confidence Interval for Mean | Lower Bound | 1.650 |  |
|  |  | Upper Bound | 2.490 |  |
|  | 5% Trimmed Mean | | 2.083 |  |
|  | Median | | 2.200 |  |
|  | Variance | | .345 |  |
|  | Std. Deviation | | .5870 |  |
|  | Minimum | | 1.0 |  |
|  | Maximum | | 2.9 |  |
|  | Range | | 1.9 |  |
|  | Interquartile Range | | .9 |  |
|  | Skewness | | -.655 | .687 |
|  | Kurtosis | | -.224 | 1.334 |
| DCAPf | Mean | | 3.790 | .1303 |
|  | 95% Confidence Interval for Mean | Lower Bound | 3.495 |  |
|  |  | Upper Bound | 4.085 |  |
|  | 5% Trimmed Mean | | 3.778 |  |
|  | Median | | 3.800 |  |
|  | Variance | | .170 |  |
|  | Std. Deviation | | .4122 |  |
|  | Minimum | | 3.3 |  |
|  | Maximum | | 4.5 |  |
|  | Range | | 1.2 |  |
|  | Interquartile Range | | .6 |  |
|  | Skewness | | .648 | .687 |
|  | Kurtosis | | -.529 | 1.334 |

| **Tests of Normality** | | | | | | |
| --- | --- | --- | --- | --- | --- | --- |
|  | Kolmogorov-Smirnov^a^ | | | Shapiro-Wilk | | |
|  | Statistic | df | Sig. | Statistic | df | Sig. |
| DCEAPf | .100 | 10 | .200^*^ | .983 | 10 | .981 |
| TDAPf | .156 | 10 | .200^*^ | .946 | 10 | .619 |
| LICAPf | .257 | 10 | .061 | .871 | 10 | .102 |
| CGAPf | .153 | 10 | .200^*^ | .947 | 10 | .630 |
| DCAPf | .195 | 10 | .200^*^ | .901 | 10 | .225 |
| *. This is a lower bound of the true significance. | | | | | | |
| a. Lilliefors Significance Correction | | | | | | |

**DCEAPf**


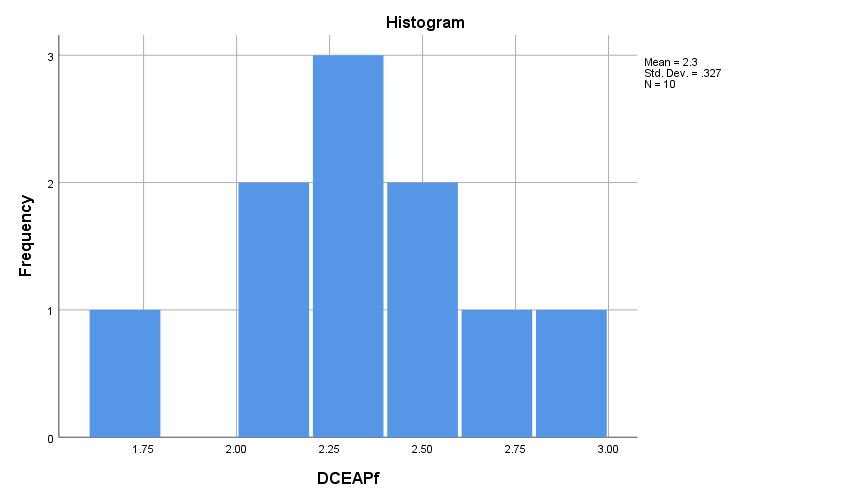

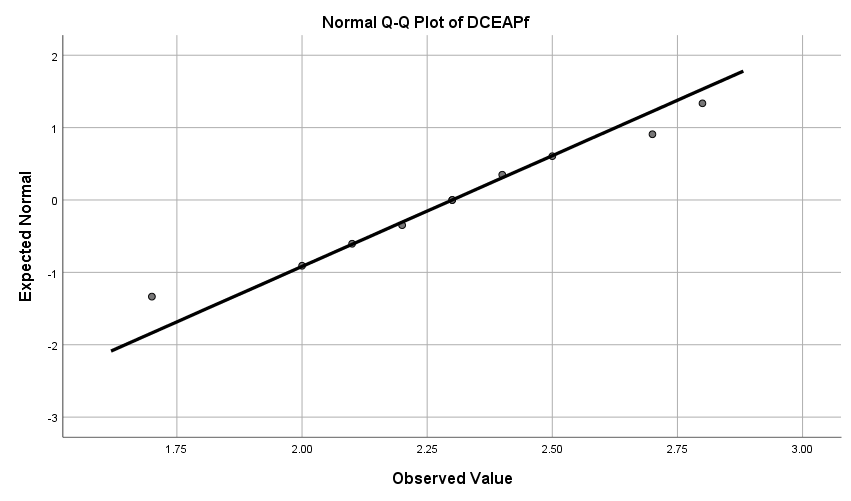


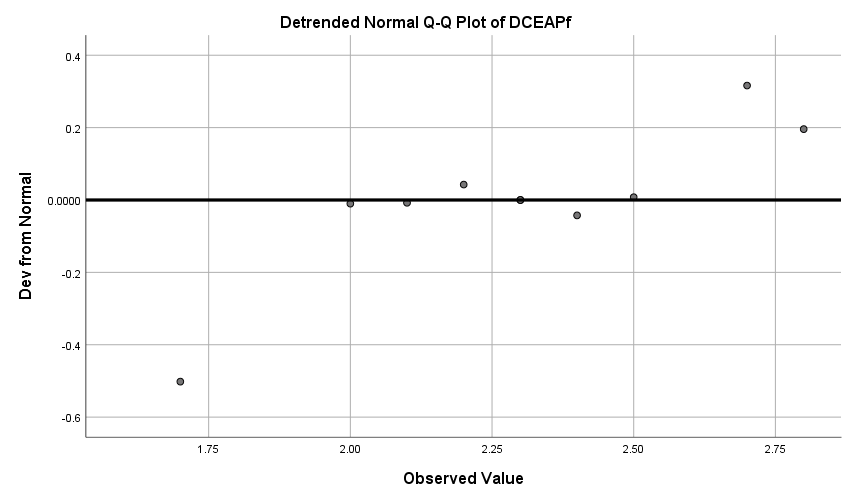

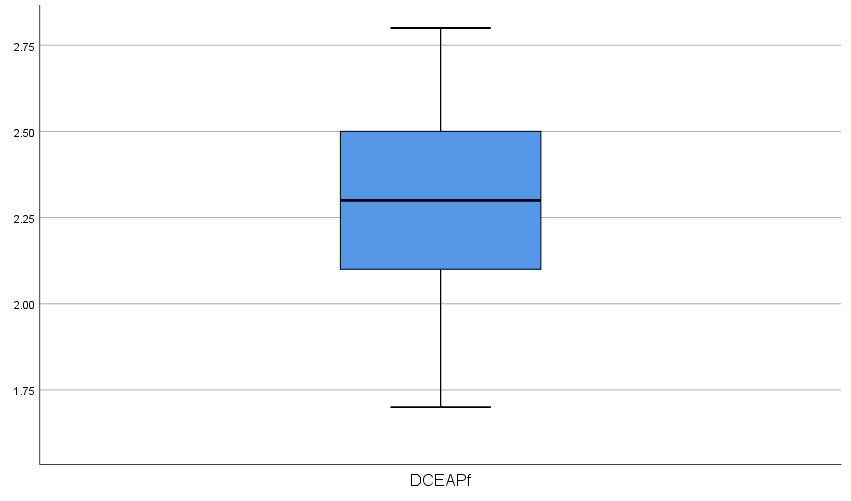


**TDAPf**


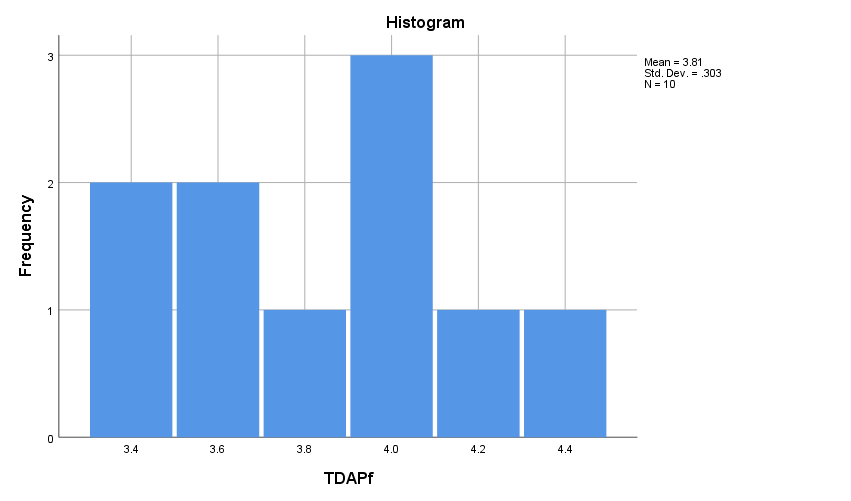

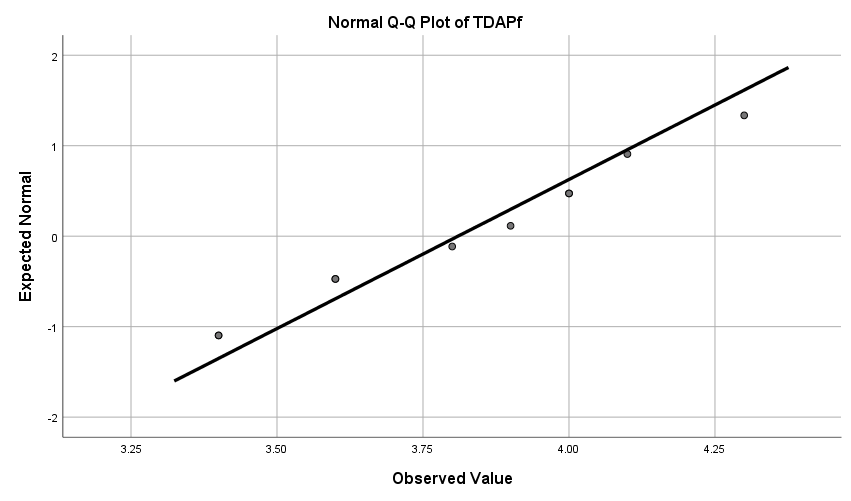


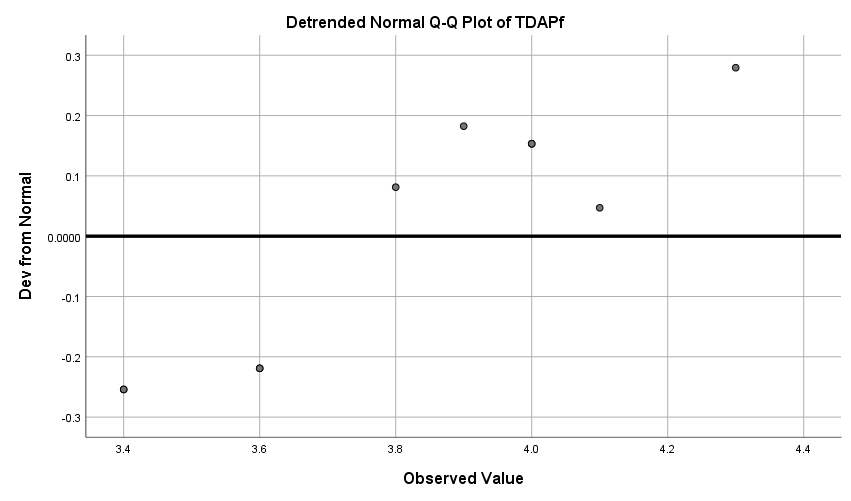

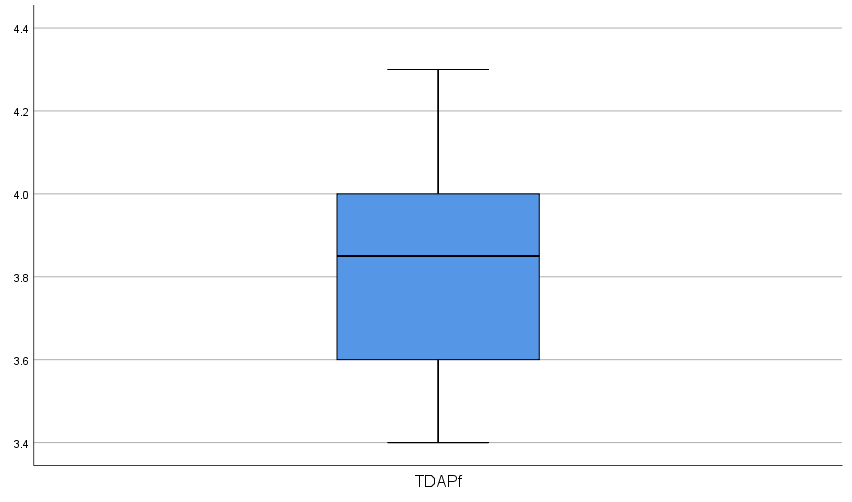


**LICAPf**


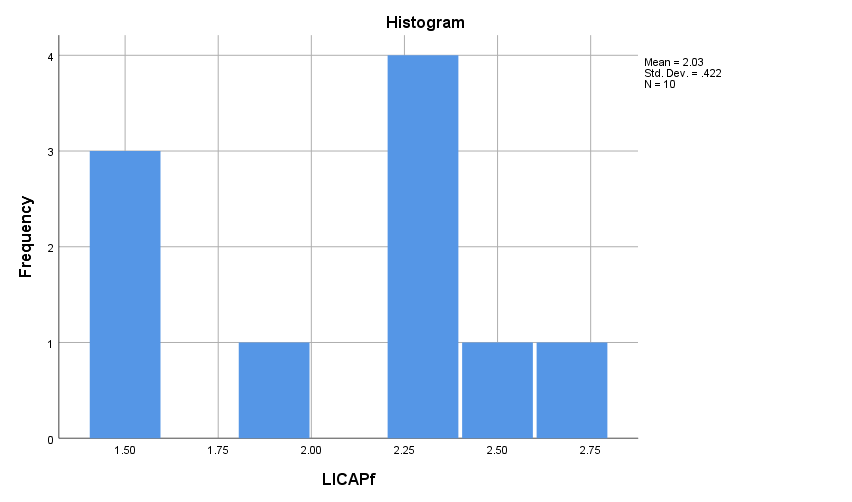

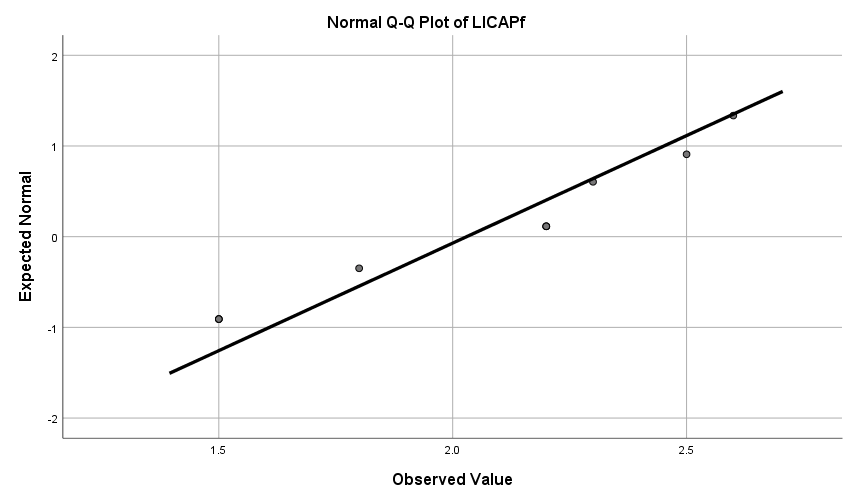


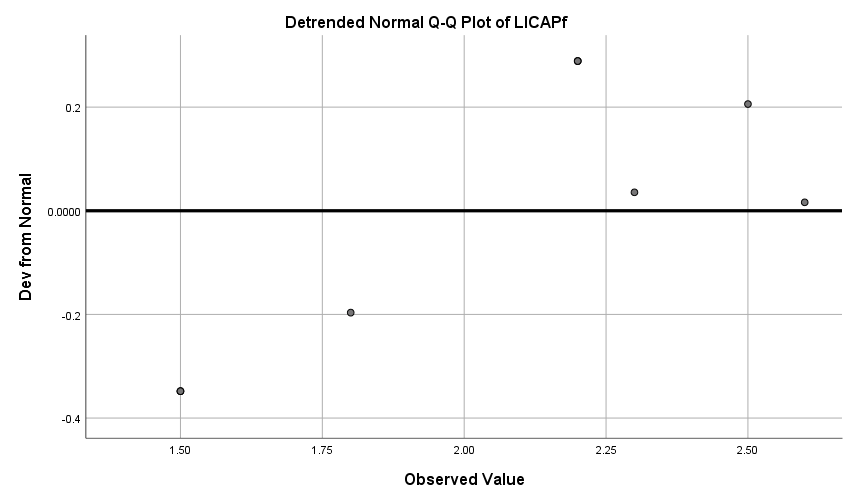

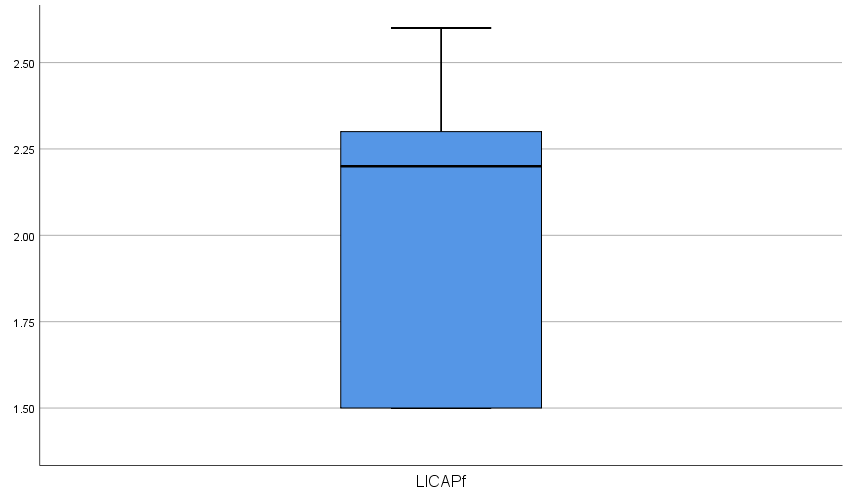


**CGAPf**


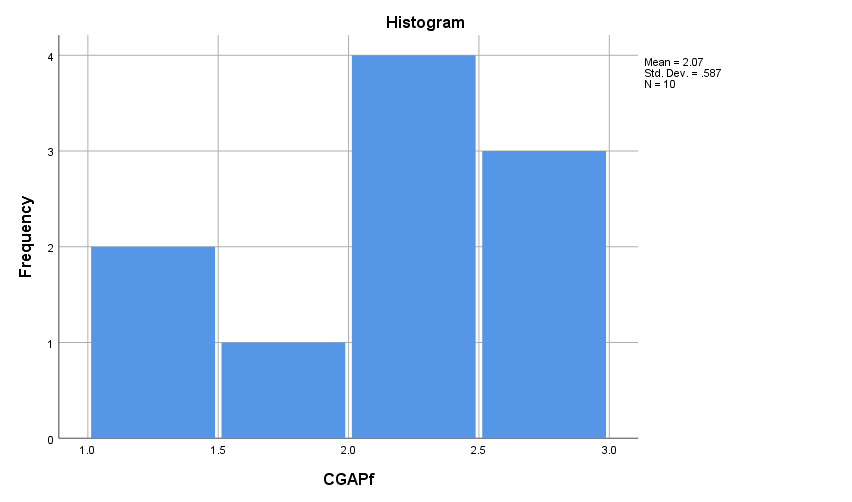

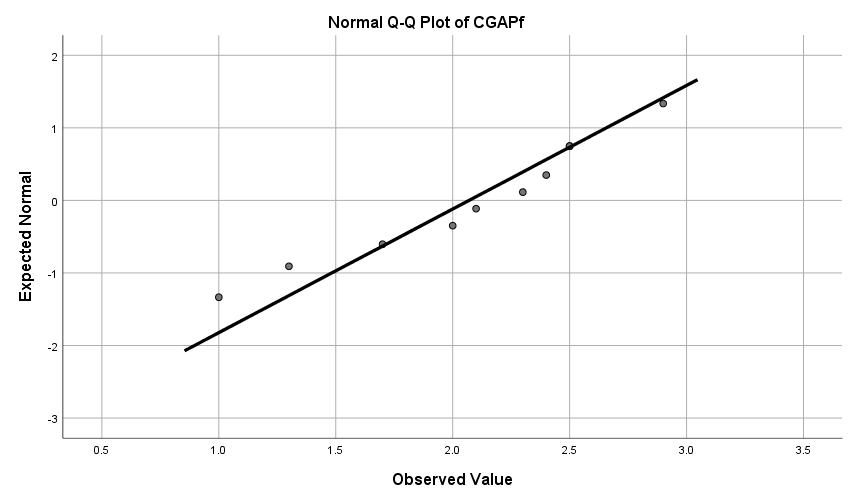


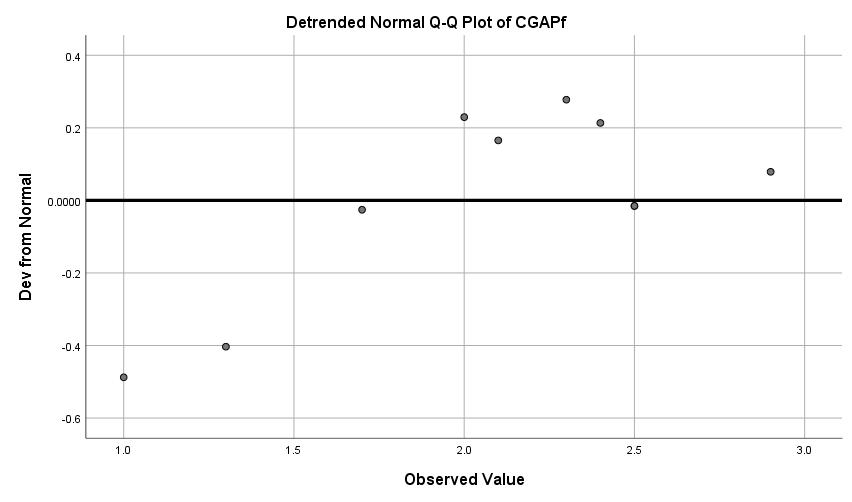

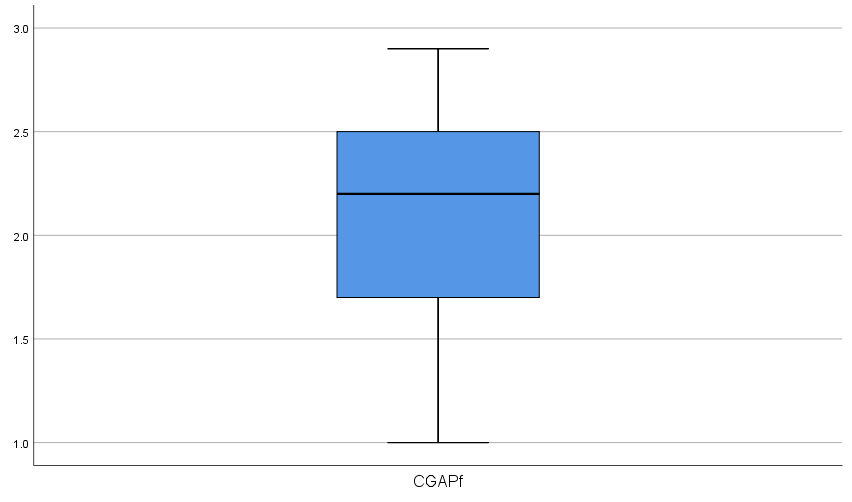


**DCAPf**


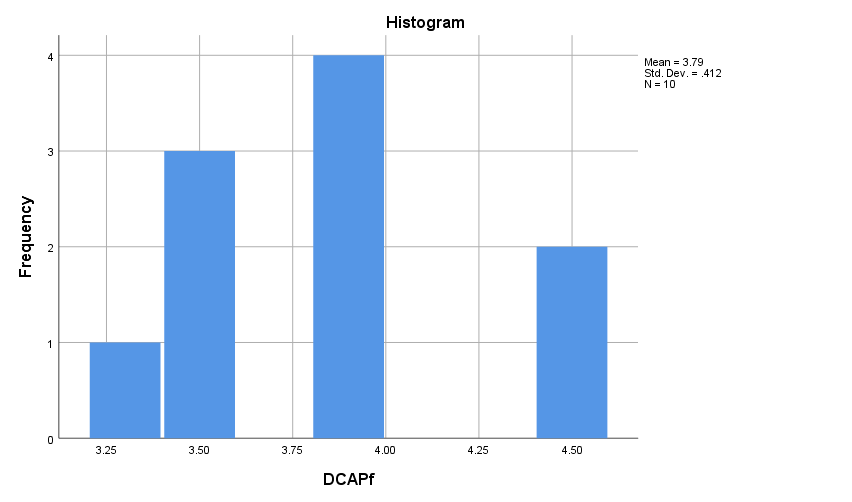

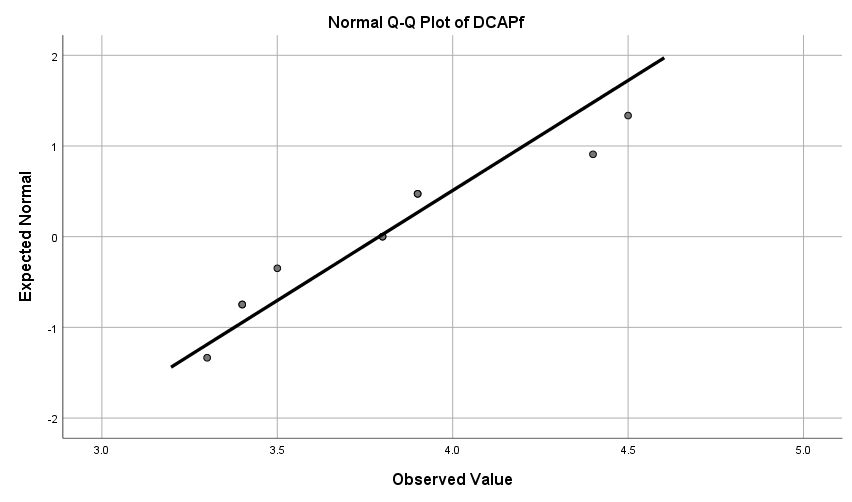


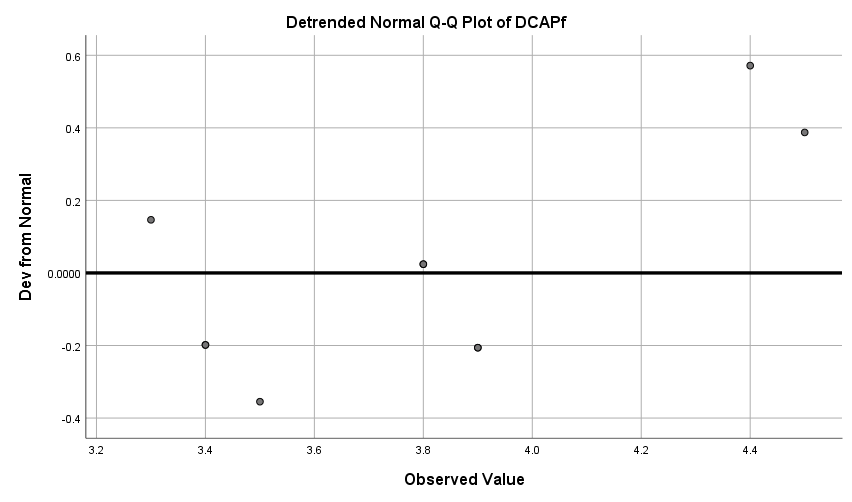

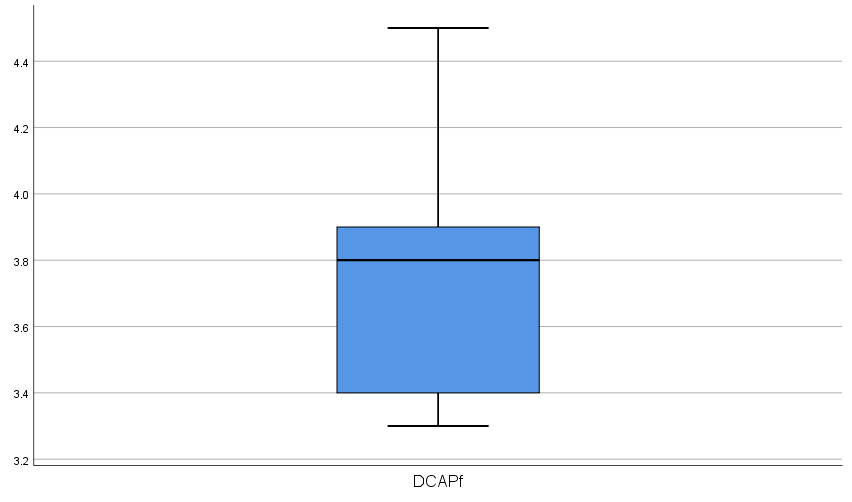


1. **Pig pedicle lenght normality test**

DESCRIPTIVES VARIABLES=DCEAPf TDAPf LICAPf CGAPf DCAPf

/STATISTICS=MEAN STDDEV MIN MAX KURTOSIS SKEWNESS.

**Conclusion**: The Skewness and Kurtosis are between -1.96 and 1.96, both Shapiro-Wilk and Kolmogorov-Smirnov tests have p-values >0.05, and the normal Q-Q plot shows a relatively normal distribution. Therefore, we conclude that, even if the sample size is small (n=10), the data is normally distributed.

**Descriptives**

| **Notes** | | |
| --- | --- | --- |
| Output Created | | 02-SEP-2021 22:22:12 |
| Comments | |  |
| Input | Data | \pig pedicle length.sav |
|  | Active Dataset | DataSet1 |
|  | Filter | <none> |
|  | Weight | <none> |
|  | Split File | <none> |
|  | N of Rows in Working Data File | 10 |
| Missing Value Handling | Definition of Missing | User defined missing values are treated as missing. |
|  | Cases Used | All non-missing data are used. |
| Syntax | | DESCRIPTIVES VARIABLES=DCEAPf TDAPf LICAPf CGAPf DCAPf  /STATISTICS=MEAN STDDEV MIN MAX KURTOSIS SKEWNESS. |
| Resources | Processor Time | 00:00:00.00 |
|  | Elapsed Time | 00:00:00.02 |

| **Descriptive Statistics** | | | | | | | | | |
| --- | --- | --- | --- | --- | --- | --- | --- | --- | --- |
|  | N | Minimum | Maximum | Mean | Std. Deviation | Skewness | | Kurtosis | |
|  | Statistic | Statistic | Statistic | Statistic | Statistic | Statistic | Std. Error | Statistic | Std. Error |
| DCEAPf | 10 | 8.2 | 13.3 | 11.330 | 1.8762 | -.620 | .687 | -1.312 | 1.334 |
| TDAPf | 10 | 5.1 | 12.0 | 8.220 | 2.1478 | .742 | .687 | .037 | 1.334 |
| LICAPf | 10 | 1.7 | 3.4 | 2.580 | .5903 | -.109 | .687 | -1.192 | 1.334 |
| CGAPf | 10 | 4.2 | 9.0 | 6.420 | 1.5411 | .100 | .687 | -.894 | 1.334 |
| DCAPf | 10 | 3.4 | 11.4 | 7.730 | 2.3362 | -.223 | .687 | .160 | 1.334 |
| Valid N (listwise) | 10 |  |  |  |  |  |  |  |  |

EXAMINE VARIABLES=DCEAPf TDAPf LICAPf CGAPf DCAPf

/PLOT BOXPLOT HISTOGRAM NPPLOT

/COMPARE GROUPS

/STATISTICS DESCRIPTIVES

/CINTERVAL 95

/MISSING LISTWISE

/NOTOTAL.

**Explore**

| **Notes** | | |
| --- | --- | --- |
| Output Created | | 02-SEP-2021 22:22:35 |
| Comments | |  |
| Input | Data | pig pedicle length.sav |
|  | Active Dataset | DataSet1 |
|  | Filter | <none> |
|  | Weight | <none> |
|  | Split File | <none> |
|  | N of Rows in Working Data File | 10 |
| Missing Value Handling | Definition of Missing | User-defined missing values for dependent variables are treated as missing. |
|  | Cases Used | Statistics are based on cases with no missing values for any dependent variable or factor used. |
| Syntax | | EXAMINE VARIABLES=DCEAPf TDAPf LICAPf CGAPf DCAPf  /PLOT BOXPLOT HISTOGRAM NPPLOT  /COMPARE GROUPS  /STATISTICS DESCRIPTIVES  /CINTERVAL 95  /MISSING LISTWISE  /NOTOTAL. |
| Resources | Processor Time | 00:00:03.02 |
|  | Elapsed Time | 00:00:02.63 |

| **Case Processing Summary** | | | | | | |
| --- | --- | --- | --- | --- | --- | --- |
|  | Cases | | | | | |
|  | Valid | | Missing | | Total | |
|  | N | Percent | N | Percent | N | Percent |
| DCEAPf | 10 | 100.0% | 0 | 0.0% | 10 | 100.0% |
| TDAPf | 10 | 100.0% | 0 | 0.0% | 10 | 100.0% |
| LICAPf | 10 | 100.0% | 0 | 0.0% | 10 | 100.0% |
| CGAPf | 10 | 100.0% | 0 | 0.0% | 10 | 100.0% |
| DCAPf | 10 | 100.0% | 0 | 0.0% | 10 | 100.0% |

| **Descriptives** | | | | |
| --- | --- | --- | --- | --- |
|  | | | Statistic | Std. Error |
| DCEAPf | Mean | | 11.330 | .5933 |
|  | 95% Confidence Interval for Mean | Lower Bound | 9.988 |  |
|  |  | Upper Bound | 12.672 |  |
|  | 5% Trimmed Mean | | 11.394 |  |
|  | Median | | 12.250 |  |
|  | Variance | | 3.520 |  |
|  | Std. Deviation | | 1.8762 |  |
|  | Minimum | | 8.2 |  |
|  | Maximum | | 13.3 |  |
|  | Range | | 5.1 |  |
|  | Interquartile Range | | 3.1 |  |
|  | Skewness | | -.620 | .687 |
|  | Kurtosis | | -1.312 | 1.334 |
| TDAPf | Mean | | 8.220 | .6792 |
|  | 95% Confidence Interval for Mean | Lower Bound | 6.684 |  |
|  |  | Upper Bound | 9.756 |  |
|  | 5% Trimmed Mean | | 8.183 |  |
|  | Median | | 7.550 |  |
|  | Variance | | 4.613 |  |
|  | Std. Deviation | | 2.1478 |  |
|  | Minimum | | 5.1 |  |
|  | Maximum | | 12.0 |  |
|  | Range | | 6.9 |  |
|  | Interquartile Range | | 2.8 |  |
|  | Skewness | | .742 | .687 |
|  | Kurtosis | | .037 | 1.334 |
| LICAPf | Mean | | 2.580 | .1867 |
|  | 95% Confidence Interval for Mean | Lower Bound | 2.158 |  |
|  |  | Upper Bound | 3.002 |  |
|  | 5% Trimmed Mean | | 2.583 |  |
|  | Median | | 2.500 |  |
|  | Variance | | .348 |  |
|  | Std. Deviation | | .5903 |  |
|  | Minimum | | 1.7 |  |
|  | Maximum | | 3.4 |  |
|  | Range | | 1.7 |  |
|  | Interquartile Range | | 1.1 |  |
|  | Skewness | | -.109 | .687 |
|  | Kurtosis | | -1.192 | 1.334 |
| CGAPf | Mean | | 6.420 | .4874 |
|  | 95% Confidence Interval for Mean | Lower Bound | 5.318 |  |
|  |  | Upper Bound | 7.522 |  |
|  | 5% Trimmed Mean | | 6.400 |  |
|  | Median | | 6.350 |  |
|  | Variance | | 2.375 |  |
|  | Std. Deviation | | 1.5411 |  |
|  | Minimum | | 4.2 |  |
|  | Maximum | | 9.0 |  |
|  | Range | | 4.8 |  |
|  | Interquartile Range | | 2.8 |  |
|  | Skewness | | .100 | .687 |
|  | Kurtosis | | -.894 | 1.334 |
| DCAPf | Mean | | 7.730 | .7388 |
|  | 95% Confidence Interval for Mean | Lower Bound | 6.059 |  |
|  |  | Upper Bound | 9.401 |  |
|  | 5% Trimmed Mean | | 7.767 |  |
|  | Median | | 7.950 |  |
|  | Variance | | 5.458 |  |
|  | Std. Deviation | | 2.3362 |  |
|  | Minimum | | 3.4 |  |
|  | Maximum | | 11.4 |  |
|  | Range | | 8.0 |  |
|  | Interquartile Range | | 3.0 |  |
|  | Skewness | | -.223 | .687 |
|  | Kurtosis | | .160 | 1.334 |

| **Tests of Normality** | | | | | | |
| --- | --- | --- | --- | --- | --- | --- |
|  | Kolmogorov-Smirnov^a^ | | | Shapiro-Wilk | | |
|  | Statistic | df | Sig. | Statistic | df | Sig. |
| DCEAPf | .239 | 10 | .109 | .869 | 10 | .097 |
| TDAPf | .222 | 10 | .176 | .906 | 10 | .257 |
| LICAPf | .153 | 10 | .200^*^ | .943 | 10 | .586 |
| CGAPf | .138 | 10 | .200^*^ | .960 | 10 | .782 |
| DCAPf | .139 | 10 | .200^*^ | .980 | 10 | .964 |
| *. This is a lower bound of the true significance. | | | | | | |
| a. Lilliefors Significance Correction | | | | | | |

**DCEAPf**


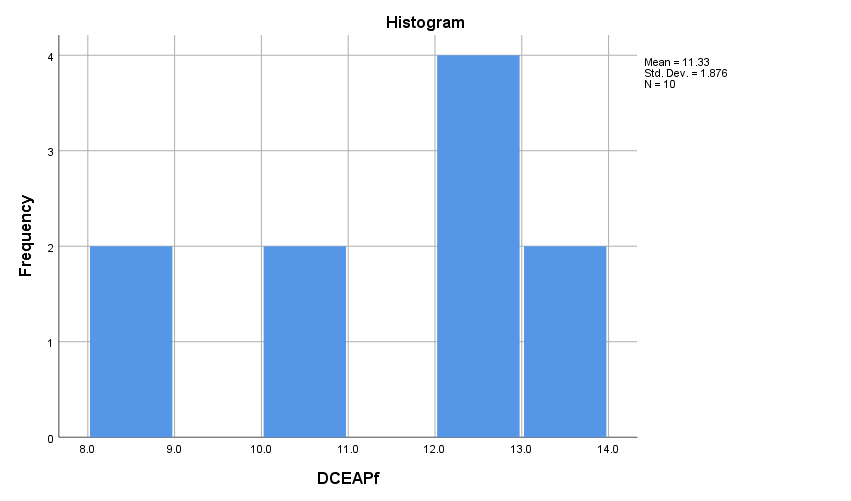

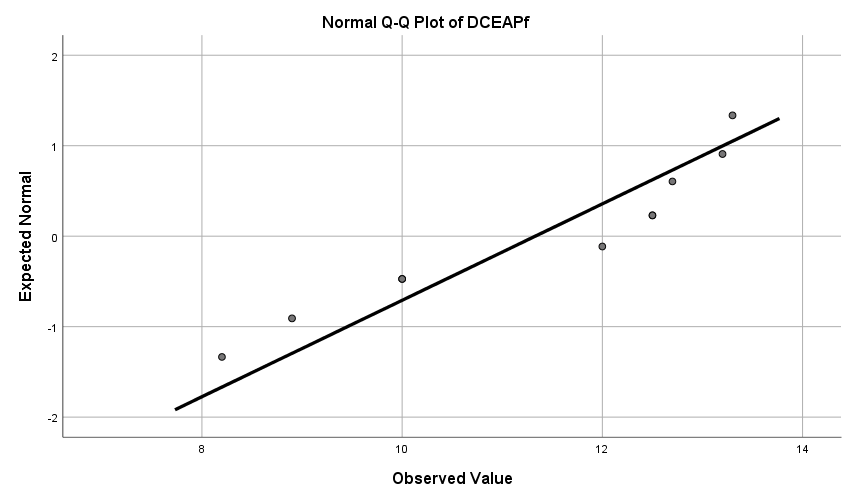


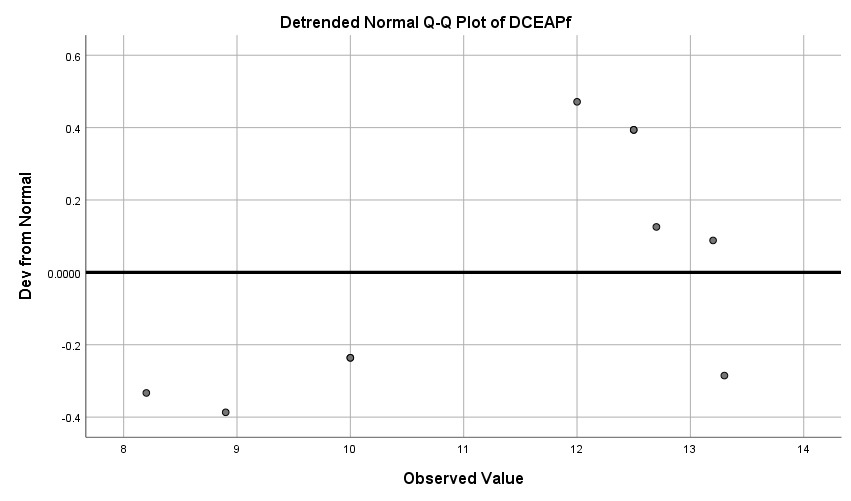

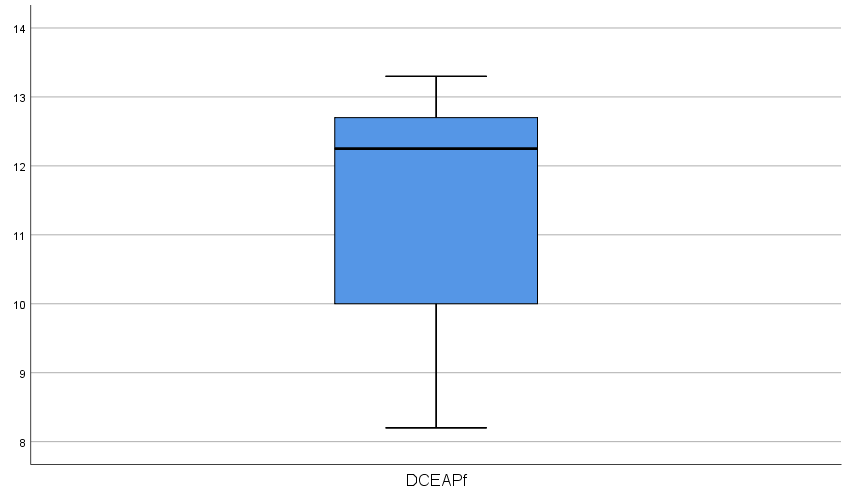


**TDAPf**


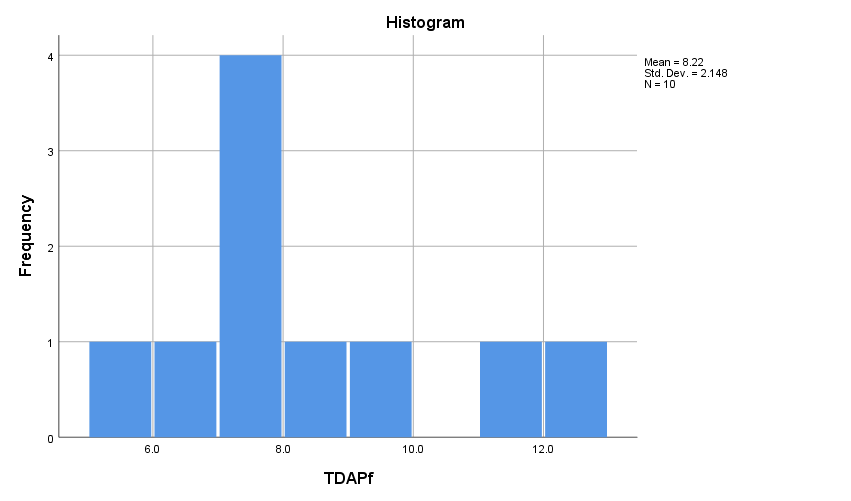

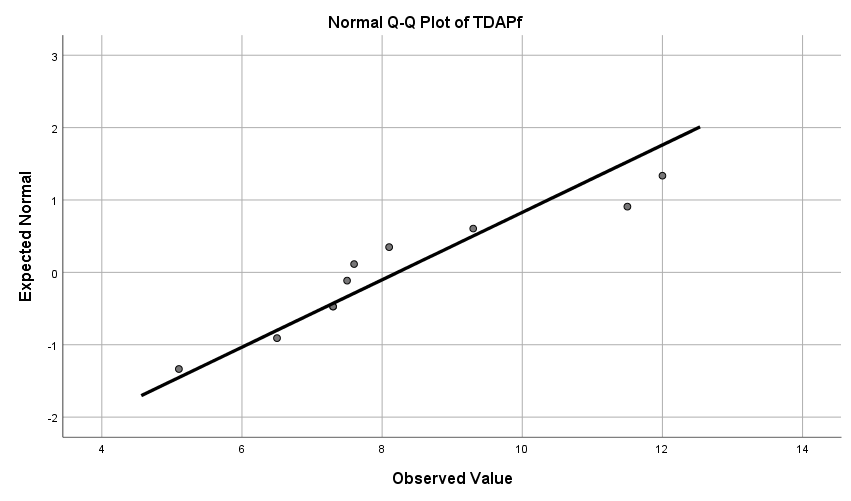


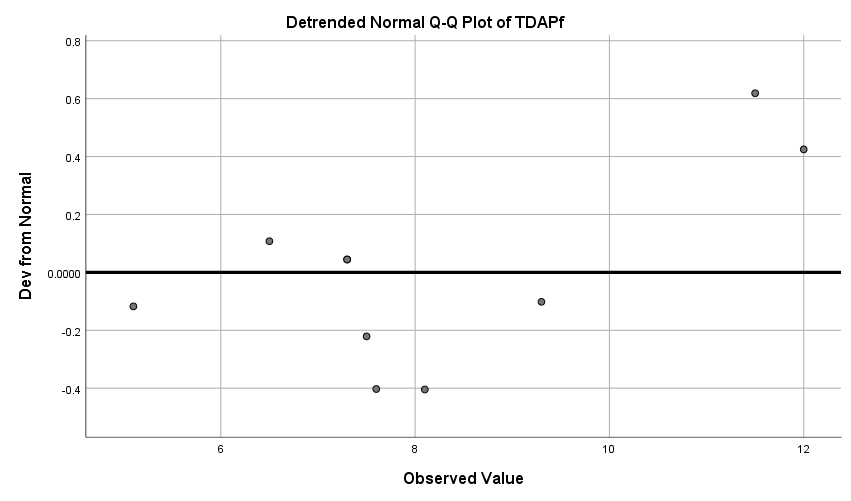

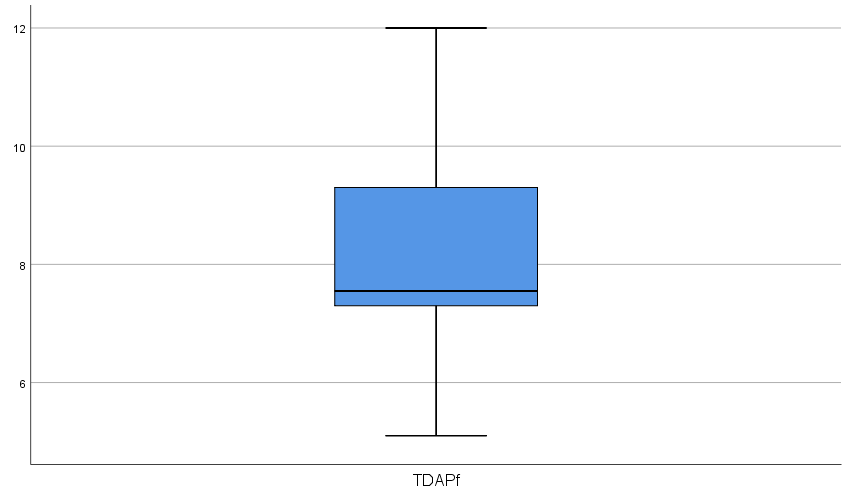


**LICAPf**


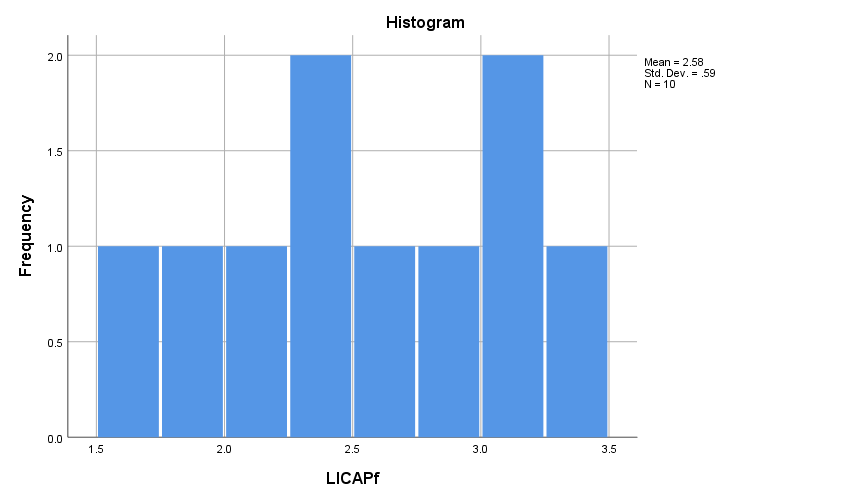

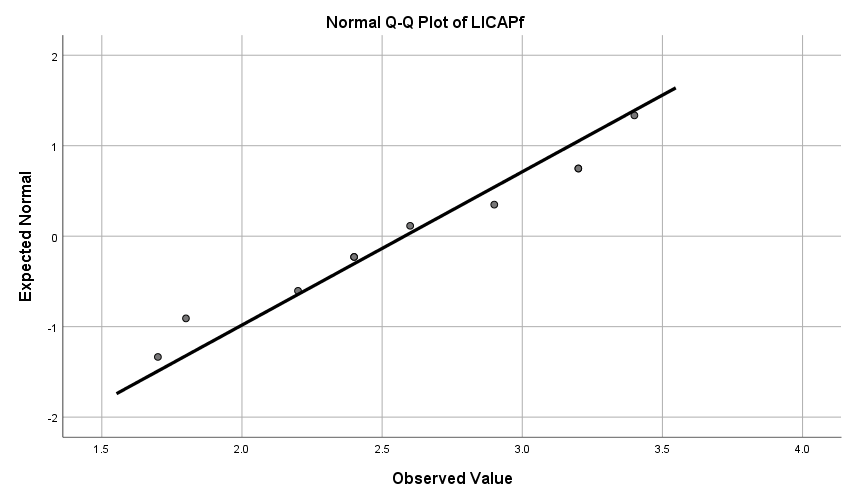


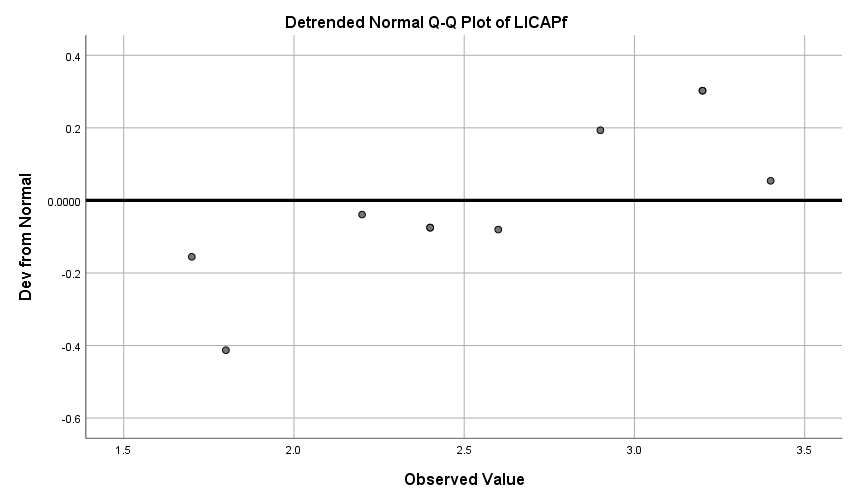

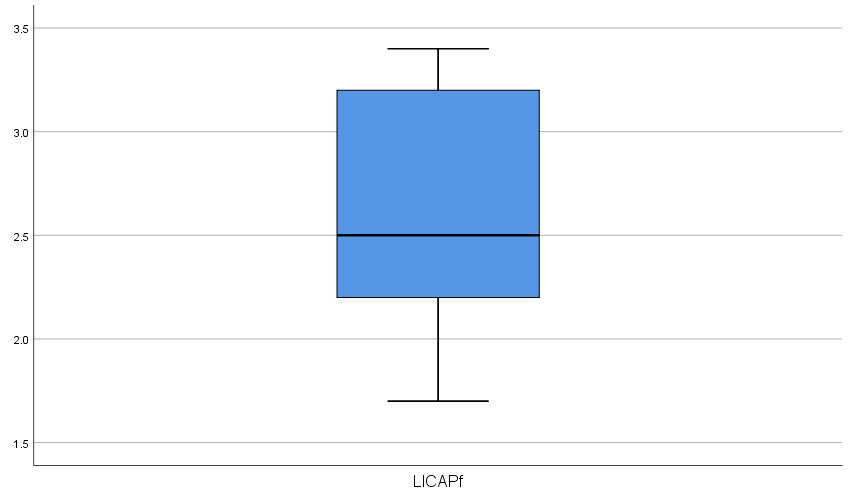


**CGAPf**


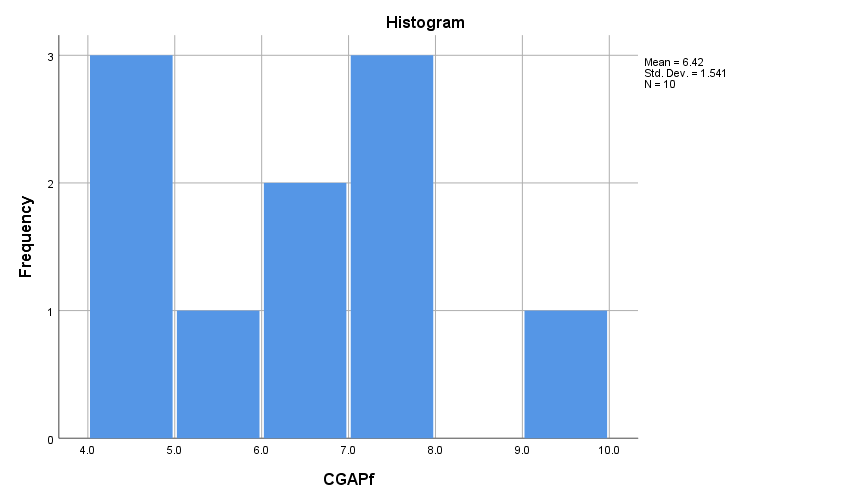

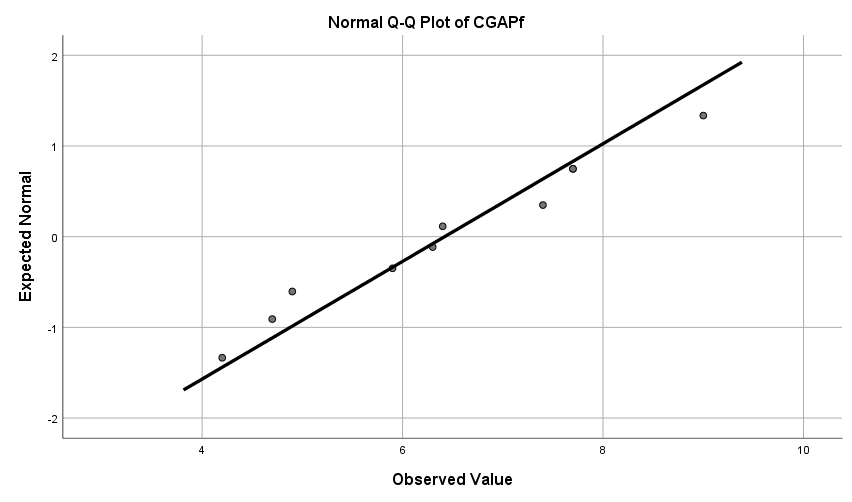


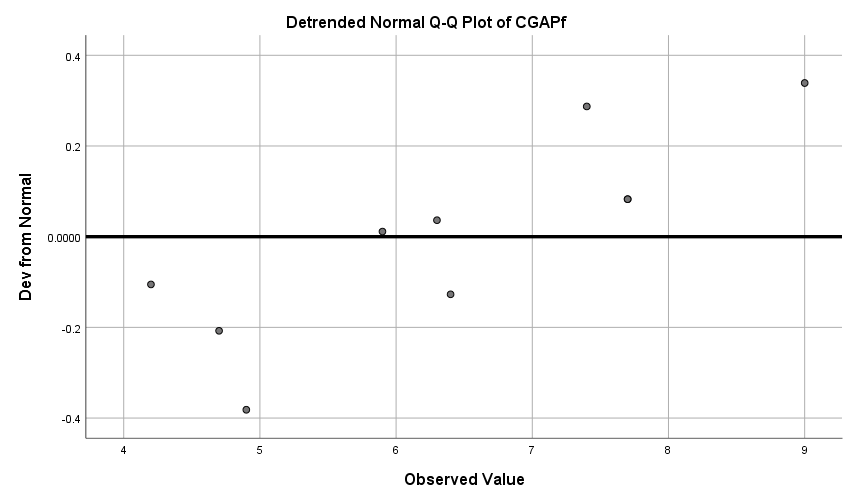

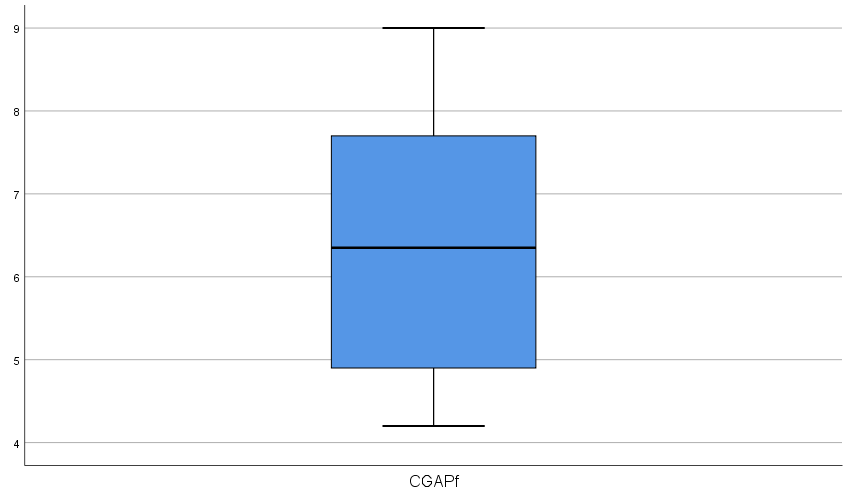


**DCAPf**


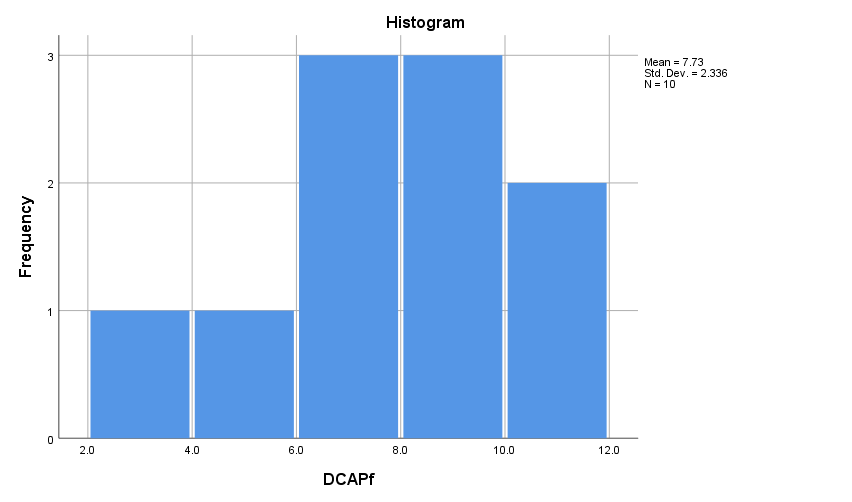

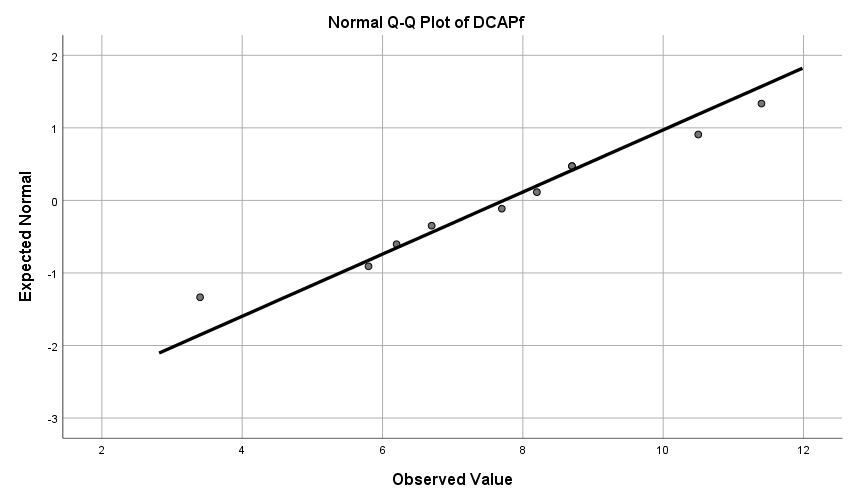


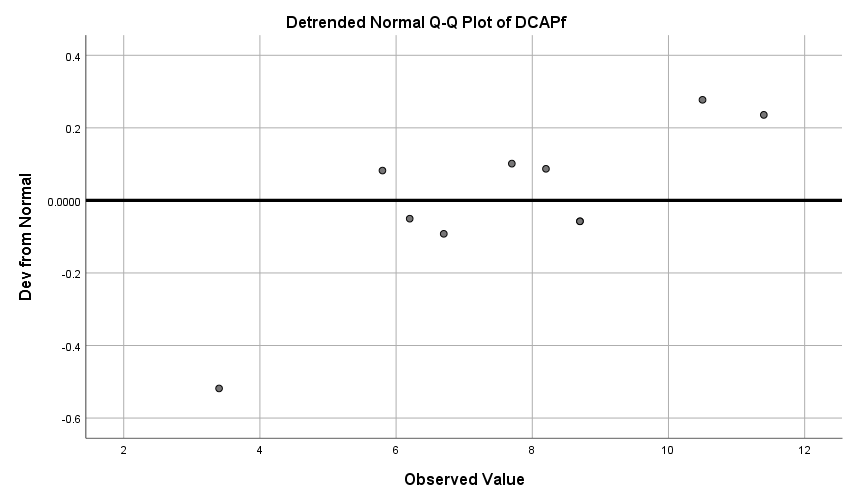

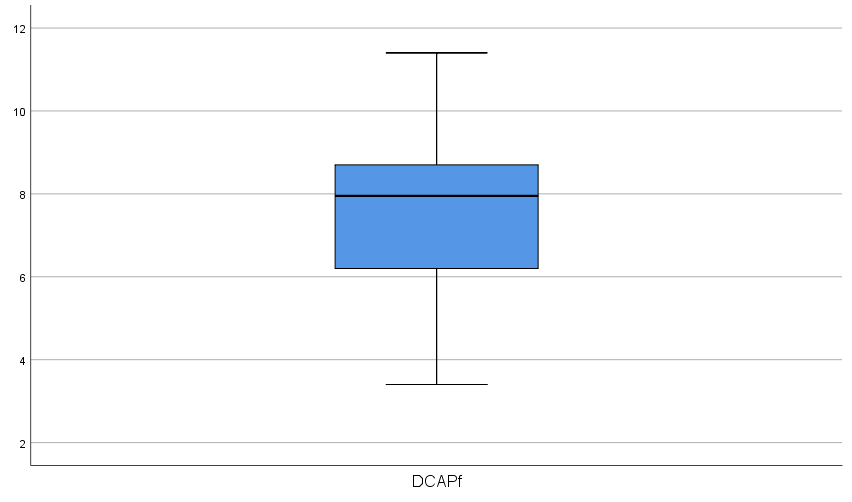

Supplement: S1 File — (DOCX) [file pone.0266873.s007.docx]
